# Postpandemic Sentinel Surveillance of Respiratory Diseases in the Context of the World Health Organization Mosaic Framework: Protocol for a Development and Evaluation Study Involving the English Primary Care Network 2023-2024

**DOI:** 10.2196/52047

**Published:** 2024-04-03

**Authors:** Xinchun Gu, Conall Watson, Utkarsh Agrawal, Heather Whitaker, William H. Elson, Sneha Anand, Ray Borrow, Anna Buckingham, Elizabeth Button, Lottie Curtis, Dominic Dunn, Alex J. Elliot, Filipa Ferreira, Rosalind Goudie, Uy Hoang, Katja Hoschler, Gavin Jamie, Debasish Kar, Beatrix Kele, Meredith Leston, Ezra Linley, Jack Macartney, Gemma L Marsden, Cecilia Okusi, Omid Parvizi, Catherine Quinot, Praveen Sebastianpillai, Vanashree Sexton, Gillian Smith, Timea Suli, Nicholas P B Thomas, Catherine Thompson, Daniel Todkill, Rashmi Wimalaratna, Matthew Inada-Kim, Nick Andrews, Victoria Tzortziou-Brown, Rachel Byford, Maria Zambon, Jamie Lopez-Bernal, Simon de Lusignan

**Affiliations:** 1 Nuffield Department of Primary Care Health Sciences University of Oxford Oxford United Kingdom; 2 Immunisation and Vaccine-Preventable Diseases Division UK Health Security Agency London United Kingdom; 3 Statistics, Modelling and Economics Department UK Health Security Agency London United Kingdom; 4 Vaccine Evaluation Unit UK Health Security Agency Manchester United Kingdom; 5 NHS England London United Kingdom; 6 Royal College of General Practitioners London United Kingdom; 7 Real-time Syndromic Surveillance Team UK Health Security Agency Birmingham United Kingdom; 8 Respiratory Virus Unit UK Health Security Agency London United Kingdom; 9 Virus Reference Department UK Health Security Agency London United Kingdom

**Keywords:** sentinel surveillance, pandemic, COVID-19, human influenza, influenza vaccines, respiratory tract infections, vaccination, World Health Organization, respiratory syncytial virus, phenotype, computerized medical record system

## Abstract

**Background:**

Prepandemic sentinel surveillance focused on improved management of winter pressures, with influenza-like illness (ILI) being the key clinical indicator. The World Health Organization (WHO) global standards for influenza surveillance include monitoring acute respiratory infection (ARI) and ILI. The WHO’s mosaic framework recommends that the surveillance strategies of countries include the virological monitoring of respiratory viruses with pandemic potential such as influenza. The Oxford-Royal College of General Practitioner Research and Surveillance Centre (RSC) in collaboration with the UK Health Security Agency (UKHSA) has provided sentinel surveillance since 1967, including virology since 1993.

**Objective:**

We aim to describe the RSC’s plans for sentinel surveillance in the 2023-2024 season and evaluate these plans against the WHO mosaic framework.

**Methods:**

Our approach, which includes patient and public involvement, contributes to surveillance objectives across all 3 domains of the mosaic framework. We will generate an ARI phenotype to enable reporting of this indicator in addition to ILI. These data will support UKHSA’s sentinel surveillance, including vaccine effectiveness and burden of disease studies. The panel of virology tests analyzed in UKHSA’s reference laboratory will remain unchanged, with additional plans for point-of-care testing, pneumococcus testing, and asymptomatic screening. Our sampling framework for serological surveillance will provide greater representativeness and more samples from younger people. We will create a biomedical resource that enables linkage between clinical data held in the RSC and virology data, including sequencing data, held by the UKHSA. We describe the governance framework for the RSC.

**Results:**

We are co-designing our communication about data sharing and sampling, contextualized by the mosaic framework, with national and general practice patient and public involvement groups. We present our ARI digital phenotype and the key data RSC network members are requested to include in computerized medical records. We will share data with the UKHSA to report vaccine effectiveness for COVID-19 and influenza, assess the disease burden of respiratory syncytial virus, and perform syndromic surveillance. Virological surveillance will include COVID-19, influenza, respiratory syncytial virus, and other common respiratory viruses. We plan to pilot point-of-care testing for group A streptococcus, urine tests for pneumococcus, and asymptomatic testing. We will integrate test requests and results with the laboratory-computerized medical record system. A biomedical resource will enable research linking clinical data to virology data. The legal basis for the RSC’s pseudonymized data extract is The Health Service (Control of Patient Information) Regulations 2002, and all nonsurveillance uses require research ethics approval.

**Conclusions:**

The RSC extended its surveillance activities to meet more but not all of the mosaic framework’s objectives. We have introduced an ARI indicator. We seek to expand our surveillance scope and could do more around transmissibility and the benefits and risks of nonvaccine therapies.

## Introduction

Prior to the COVID-19 pandemic, sentinel surveillance was orientated toward influenza and its associated winter pressures [1-6]. It has subsequently evolved to include a systematic collection of acute respiratory infections (ARIs) and a wider range of indicators. The World Health Organization (WHO) Global Influenza Surveillance and Response System (GISRS) was launched in 1952 to provide a global response to influenza and other respiratory infections [7,8]. The focus of the GISRS and other national surveillance networks is seasonal influenza monitoring and associated vaccination effectiveness studies [9,10], as well as pandemic preparedness [11,12]. Virology testing became an essential component, with serosurveillance introduced into some systems [13]. Prepandemic virological testing was largely carried out in the winter season with influenza-like illness (ILI) as the key clinical indicator of community influenza infection [14]. In addition to ILI, ARI started to be used by the European Centre for Disease Prevention and Control as a surveillance indicator [15,16]. The WHO 2013 global epidemiological standards for influenza surveillance proposed the use of severe ARI (SARI) as an indicator. SARI is defined as an incident ARI in a person admitted to a hospital [17,18]. The WHO mosaic framework suggests sentinel ILI, ARI, and SARI surveillance as the core approach for monitoring the epidemiological characteristics of respiratory viruses in interpandemic periods [16].

Subsequent to the COVID-19 pandemic, the WHO published its mosaic framework for respiratory disease surveillance [16,19]. It recommended that the surveillance strategies of countries include the virological monitoring of influenza, SARS-CoV-2, respiratory syncytial virus (RSV), and other viruses with pandemic potential. The mosaic has a broad framework and includes 14 surveillance objectives set out across three domains: (1) detection and assessment of respiratory viruses; (2) monitoring their epidemiological characteristics; and (3) informing on the use of health interventions [16,19].

The Royal College of General Practitioners (RCGP) has been collecting data about respiratory and other infections in England in its epidemic research center since 1957 [20]. This research center became rebranded as the RCGP Research and Surveillance Centre (RSC) and has been conducting sentinel surveillance since 1967 [21], in collaboration with the UK Health Security Agency (UKHSA) and its predecessor bodies. The RSC has included reference laboratory virology since the 1993-1994 season [22]. The network has grown to almost 2000 practices in England and Wales (31.6% of the active practices) with a contemporary extract of over 19 million patients (31.9% of the England and Wales population) in 2023 [23].

This protocol describes the Oxford-RCGP RSC’s plans for the 2023-2024 sentinel surveillance season and evaluates them against the WHO mosaic framework. The RSC will be offering all-year-round virology and sentinel surveillance of respiratory infections in collaboration with the UKHSA. Our extended surveillance includes the adoption of SARI as an important severity indicator, alongside ILI and other components of the WHO mosaic framework.

The objectives are as follows: (1) describe the planned patient and public involvement (PPI) with the RSC, with the aims of improving public understanding of the RSC’s program and co-designing changes to our sentinel surveillance; (2) develop an ARI digital phenotype and contemporaneously report the incidence of ARI and SARI, including new severity indicators, using primary care data; (3) collect and share high-quality data to support vaccine effectiveness (VE) studies for COVID-19 and influenza vaccines in the coming season and enable the reporting of RSV’s disease burden; (4) ensure that the volume of virology and serology sampling from member practices following our sampling framework is sufficient to determine VE by vaccine type and has the minimum required clinical data recorded; (5) introduce technological developments by using general practitioner (GP) and laboratory links to support virology and serology sampling, establishing a messaging system to enable more representative sampling and targeted sampling when required, increasing point-of-care testing (POCT) capability, piloting virology sampling from asymptomatic individuals, and testing for pneumococcus infection; (6) create a biomedical resource that provides a unique longitudinal clinical resource and enables genomic surveillance by linking individual-level human phenotypes to the genomic sequences of viruses detected in those individuals; and (7) describe the legal basis and governance framework for conducting sentinel surveillance.

## Methods

### Comparison With the WHO Mosaic Framework

We describe our approach to sentinel surveillance in functional components. Many of these, such as PPI, span across all of the mosaic’s sentinel objectives. Others sit outside or beyond its scope, for example, bacterial causes of infection and information governance requirements. We conclude the results section with a table summarizing the surveillance objectives achieved and those to be delivered beyond its scope.

### PPI Group

We will use 2 channels of PPI and engagement within the RSC. The first channel is with national PPI groups, the Health Data Research-UK and the RCGP Patient and Carer Participation Groups. Additional national patient representatives will be recruited from the People in Research portal of the National Institute for Health and Care Research [24] to ensure geographical representation across England. The second channel includes local patient participation groups of general practice members of the RSC network. Opportunities for patients to provide feedback will be communicated to all network practices in England.

We will invite patient representatives to participate in 2 meetings per year held with the UKHSA and Oxford-RCGP RSC. The meetings aim to raise awareness of surveillance, including its use of patient data, and to gain input on the RSC’s work, particularly on optimizing the acceptability of sampling and the feedback of results. PPI feedback will be used mainly to improve communication with patients, but may inform other areas of the surveillance. We will send a monthly newsletter covering topics and findings related to surveillance to increase transparency and patient engagement (Multimedia Appendix 1). We will use the cube framework to plan and evaluate PPI [25]. To improve transparency in reporting PPI and its impact, we will use a standard international guideline (GRIPP2 checklist) [26].

### ARI Phenotype

A digital phenotype is a set of rules that allows the identification of cases, such as ILI or ARI, from the computerized medical record (CMR) [27]. At the RSC, we have a well-established digital phenotype for ILI. To bring our strategy in line with the most recent WHO recommendations, we are developing a new ARI phenotype for the coming season.

The phenotype is being built around Systematized Nomenclature of Medicine (SNOMED) Clinical Terms (CT) as this is the medical terminology mandated by the National Health Service (NHS) and used by all primary care providers [28]. The phenotype consists of code lists representing common conditions that make up the ARI concept. This allows the identification of ARI events coded by member practices in the CMR.

We plan to build on the ARI phenotype by characterizing the nature and severity of ARI events. We will do this by exploring primary care codes recorded in association with ARI and by linking the primary care data to secondary care records [29,30]. We differentiate ARI from SARI by checking whether ARI leads to hospitalization, as recorded in the CMR.

**Figure 1:**
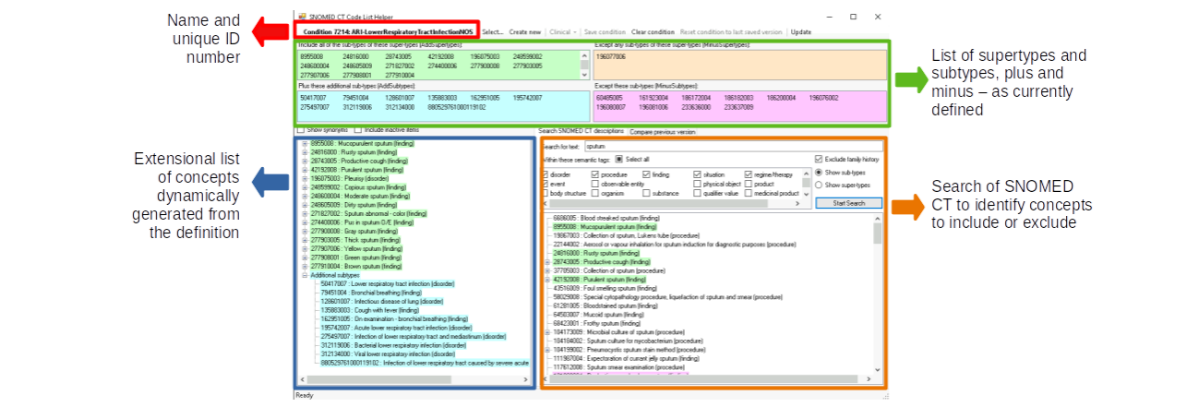
Tool used to facilitate the intentional identification of Systematized Nomenclature of Medicine Clinical Terms (SNOMED CT) refsets. The lower figure demonstrates how SNOMED CT supertypes and subtypes are included or excluded using the Helper Tool.

Our phenotype development uses the Phenotype Execution Modeling Architecture (PhEMA) toolkit [31]. The Health Level-7 Fast Healthcare Interoperability Resources (FHIR) is a global standard for passing health care data between systems [31] and is used by the NHS. The PhEMA comprises developing an ontological layer using clinical query language and then presenting a “code list” using the Health Level-7 FHIR value set format or as a SNOMED CT refset. For example, the SNOMED clinical term “Lower respiratory tract infection (disorder)” (SCTID: 5041700) has 10 child codes (also known as subtypes), which are all automatically included. If extra child codes are included, they will automatically be included in our definition, unlike the extensional process where “code lists” need frequent review [32]. We have a “Helper Tool” developed in-house to facilitate the selection of SNOMED CT (Figure 1).

### High-Quality Data to Support Influenza and COVID-19 VE and Other Studies

We will collect and share high-quality data, such as primary care CMR and vaccine exposure data, to support VE studies for COVID-19 and influenza vaccines and enable the reporting of RSV’s disease burden in the coming season. We will focus on the data collection areas that need better data quality as follows: (1) Identifying and coding cases of ARI, SARI, and ILI as a problem title in CMRs, including whether these are first or new episodes [33]; (2) Consistent capturing of symptoms, signs, and any markers of severity upon the presentation of people with ARI; (3) Complete recording of vaccine exposure data, particularly for influenza and COVID-19 vaccines, but other vaccines, such as pneumococcus, may be relevant; (4) Complete recording of sociodemographic and comorbidity data; and (5) Recording of outcomes in both primary care and linked secondary care data sets. This is particularly important for people who have had virology sampling (see the next section) and practices that are incentivized through data quality payments.

We also need sufficient data (ie, sample size) to support UKHSA’s syndromic surveillance [34,35], our Weekly Return [36,37], and our Annual Report [38]. The Weekly Return and Annual Report will be in their 56th year of production. This will set out to make the network as nationally and regionally representative as possible by recruiting additional practices, particularly to improve virology sampling.

### Virology and Serology Sampling and Testing

We collect serology samples from volunteer patients who are attending their practice for a routine blood sample appointment by asking them to contribute one more blood sample to the serology surveillance. Patients are invited through messages before their routine blood tests and are provided a link for further information about the serology surveillance. Verbal consent is taken from the patients before the sampling. We aim to collect 500 serology samples per month from each of the 3 age groups (younger than 18 years, 18 to 64 years, and 65 years or older) with representative sampling across the network. We plan to divide the younger than 18 years age group based on immunization age groups in the future to allow more granular information to be collected. Serology samples are batch processed in different labs depending on operational needs, for example, understanding vaccine waning in immunocompromised people.

We collect virology samples (nasal and pharyngeal swabs) all year round from patients who present ILI or ARI symptoms to general practices and meanwhile capture their symptom onset day. We aim to reach 1000 samples per week every week in the coming year. Virological samples collected in RSC surveillance practices are analyzed in UKHSA’s respiratory virus reference laboratory in Colindale, London. Practices code the results returned from the UKHSA according to SNOMED CT or Read Code (Multimedia Appendix 2), along with the date that the swab was taken. The test results are used for virological surveillance as well as test-negative case-control studies [39] that evaluate VE for influenza and COVID-19 vaccines.

**Figure 2:**
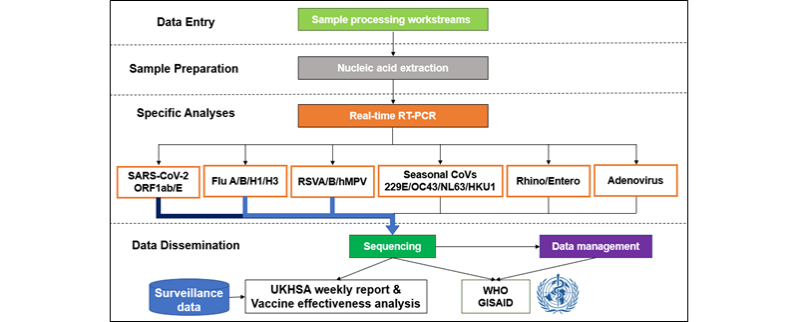
Royal College of General Practitioners Research and Surveillance Centre reference lab virological surveillance overview. GISAID: Global Initiative on Sharing All Influenza Data; RSV: respiratory syncytial virus; RT-PCR: reverse transcription polymerase chain reaction; UKHSA: UK Health Security Agency; WHO: World Health Organization.

The UKHSA tests for a panel of 8 viruses (Figure 2), including (1) SARS-CoV-2; (2) influenza (influenza A subtypes are differentiated based on their hemagglutinin [H] and neuraminidase [N] surface proteins; the 2 subtypes that are commonly in circulation are H1N1 and H3N2, though some influenza A cases are only reported as influenza A; influenza B is reported collectively, with close monitoring of circulating lineages); (3) RSV A and B; (4) human metapneumovirus (hMPV); (5) other seasonal coronaviruses (NL63, 229E, OC43, and HKU1), in addition to SARS-CoV-2; (6) adenovirus; (7) human rhinovirus; and (8) enterovirus.

To reach a sufficient volume of tests, the behavioral change we want primary care clinicians to achieve is conducting virology and serology sampling where possible and making CMR system entries of high data quality. We will use the behavioral change wheel, otherwise known as the COM-B model, to theorize behavioral change expected in clinicians. This suggests that for a behavioral change (B) to take place, an individual requires capability (C), opportunity (O), and motivation (M) [40]. We are using the COM-B model as a high-level theoretical frame to describe our interaction with practices rather than a formal experimental work.

### New Technologies and Capabilities

We have an ambitious program of introducing new technologies and extending the scope of our surveillance. We are looking to introduce 3 new technologies in the coming year: (1) LabLinks, integrating our surveillance sampling with online pathology test requests carried out via primary care CMR systems; (2) creating a POCT-ready nested cohort within the RSC; and (3) introducing a messaging system that enables us to effectively target specific groups for sampling.

The 2 surveillance capabilities we are looking to introduce are (1) urinary antigen (UAG) tests that test for pneumococcus infection, and (2) collecting virology swabs from asymptomatic infections.

We will conduct a feasibility study of testing for *Streptococcus pneumoniae* infection using UAG. We would like to perform this study over 1 to 2 years, recruiting 2 practices from each NHS region, with ARI patients having both respiratory swabs and urine tests. We will also pilot asymptomatic testing for our panel of viral tests [41]. Running this within the RSC will enable linkage of virology results to medical records and the monitoring of household transmission as we can identify people in the same household [42-44]. The protocols for the feasibility and pilot studies will be published separately.

### Creation of a Biomedical Resource

We will complete the work to create a unique biomedical resource that offers 2 unique opportunities for research. First, we will create a longitudinal database that runs back to the start of the RSC’s involvement in sentinel surveillance in 1967. Second, we will link clinical records and the virology results for all tests performed at UKHSA’s reference laboratory. This will create a resource to support the emerging discipline of genomic surveillance [45,46] by linking clinical phenotype, as defined in an individual’s CMR, at the individual level with details of the infecting virus, including its genome sequence. Summary results of the unique longitudinal data are presented.

### Ethical Considerations

Pseudonymized primary care data and samples from general practices collected for surveillance purposes are processed under Regulation 3 of the Health Service (Control of Patient Information Regulations 2002) and annually renewed under Regulation 7 by UKHSA’s Caldicott Guardian [47]. Any further research or studies require their own ethical approval and approval of the Joint RSC Committee of the University of Oxford and RCGP.

There are data-sharing agreements in place with every GP practice within the network, where the purpose of data collection and the processing of activities are stated. We link primary care data collected via sentinel surveillance to secondary care data provided by NHS England via a bespoke data sharing agreement that is renewed annually. All the data controllers (UKHSA, RCGP, and University of Oxford) complete NHS England’s data security and protection toolkit to meet the performance standards set by the National Data Guardian [48].

## Results

### PPI Group

We presented the protocol to patient representatives in July 2023. Patient representatives proposed promoting self-testing for virology to help increase the uptake of sampling. They also suggested considering swabbing in different settings, including a pharmacy, as patients with respiratory illnesses may be more likely to visit a pharmacy rather than a GP.

The patient groups have suggested that the following themes and content should form part of our PPI communications: (1) information about how patient data are used in surveillance, (2) information about how sentinel surveillance fits in with pandemic preparedness, and (3) further personal areas of interest, for example, to better understand patterns in patients with post–COVID-19 condition. The PPI representatives expressed that some patients may have concerns about patient confidentiality and data use, which can be overcome by written dissemination and regular engagement.

Based on initial feedback, we have tailored our communication to focus on key areas of interest to the public. For example, we have delivered a presentation and discussion group to patients at a Midlands practice, which focused on how data are used for surveillance and how transparency can be improved. We have developed a poster designed for practice waiting rooms to promote awareness of the RSC. We consider patient feedback in training our surveillance practices (eg, we inform new practices that patients like to receive their swab results, which may enhance patients’ experience of care).

### ARI Phenotype

**Figure 3:**
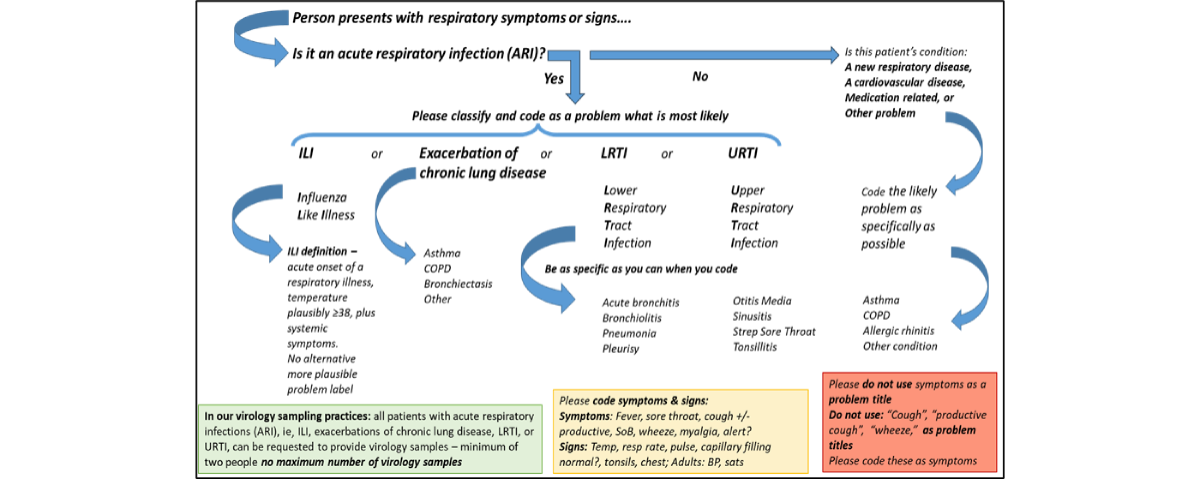
Research and Surveillance Centre recommendations for classifying the etiology of respiratory symptoms or signs in people presenting to primary care. COPD: chronic obstructive pulmonary disease; ILI: influenza-like illness; LRTI: lower respiratory tract infection; SoB: shortness of breath; URTI: upper respiratory tract infection.

To ensure that we identify and classify all relevant coded events in the CMR, the ARI phenotype will be hierarchical. The top level will represent the overall ARI concept. The middle level will include codes for upper respiratory tract infection (URTI), lower respiratory tract infection (LRTI), exacerbations of chronic lung disease, and ILI. The bottom level will include codes for lower-level syndromes, such as sinusitis, that are descendants of middle-level syndromes (Figure 3). We will recommend that RSC network member practices consider coding ARI-associated diseases and symptoms as the diagnosis when patients present with ARI symptoms.

**Figure 4:**
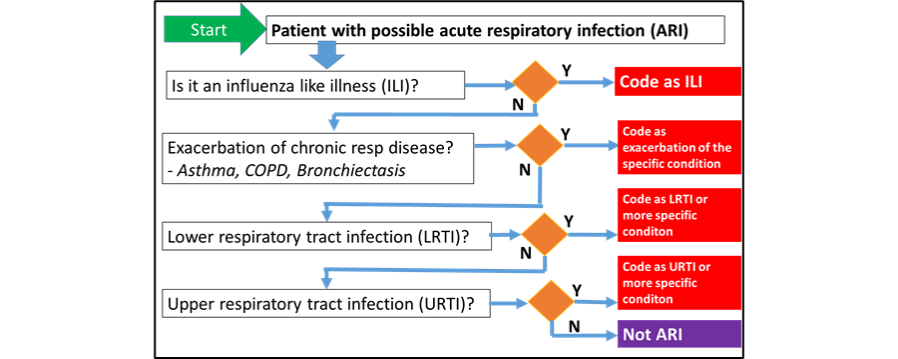
Research and Surveillance Centre recommendations for classifying possible acute respiratory infection presentation to primary care. The coding sequence is to promote improved recording of influenza-like illness cases and exacerbations of chronic respiratory disease. COPD: chronic obstructive pulmonary disease.

We have also developed a recommended coding sequence for patients presenting with ARI to primary care. The overall ARI and ILI signals are important for respiratory surveillance; hence, we recommend considering ILI first, using the RSC’s ILI definition: ARI with measured or clinically plausible temperature ≥38 °C, cough, systemic upset, such as headache or myalgia, sudden onset, and absence of a more plausible diagnosis. Our current surveillance categories do not reliably capture exacerbations of chronic lung diseases, which are vulnerable to severe outcomes from infective exacerbations [49,50]. We are therefore asking practices to consider this next. We then ask the consulting clinician to consider LRTI and subsequently URTI (Figure 4).

We are also standardizing the recording of symptoms, signs, and any emergency management decisions made for ARI events, so we request RSC practices to record these in the CMR. We fully understand the pressures on consulting time, so we will be producing data entry forms that practices can use in the major brands of CMR systems to facilitate high-quality data entry. The key symptom data to be coded are as follows: (1) date of onset of symptoms; (2) presence of “absence of fever” because infections, particularly in older people, may not be associated with fever [51]; (3) sore throat symptoms; (4) cough or no cough, and if coughing, is it productive; (5) coryza and nasal symptoms; and (6) presence or absence of shortness of breath and wheezing. The signs we would like to see coded are as follows: (1) measured tympanic temperature; (2) peripheral oxygen saturation, where available; (3) pulse rate; (4) respiratory rate; (5) upper respiratory signs where present, including cervical and anterior cervical lymphadenopathy, and any tonsillar exudate or enlargement; and (6) lower respiratory signs, including wheezing or other physical signs.

### High-Quality Data to Support VE and Other Studies

**Figure 5:**
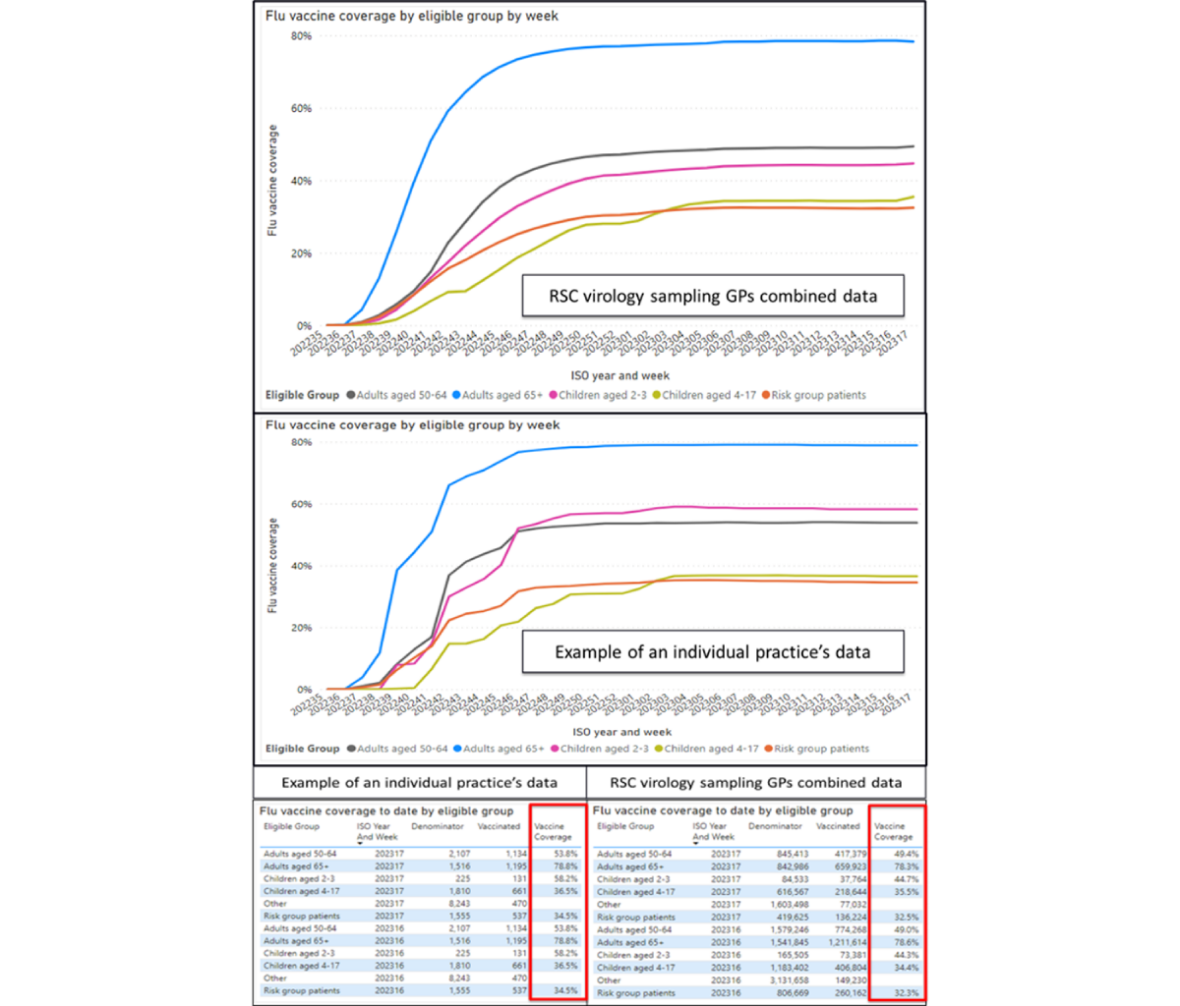
Vaccine exposure data, taken from our observatory displaying graphically as well as numerically the practice levels of vaccine uptake (2022-2023 season). GP: general practitioner; RSC: Research and Surveillance Centre.

To enhance data quality, RSC member practices will receive feedback about their recording of key sociodemographic variables, vaccine exposure, and risk groups. We also provide a dashboard to enable RSC practices to compare their rate of vaccination with the rest of the network (Figure 5).

Most of the relevant data in VE studies are recorded as part of standard care, but some are not recorded to a satisfactory standard. For example, some important sociodemographic data are not automatically recorded, including ethnicity, smoking status, and obesity. Vaccine exposure data should include brand and batch wherever possible, but these recordings are problematic for vaccinations outside general practices.

Risk groups and patient outcomes are also important for VE and other studies, and most of these data are recorded well as part of chronic disease management. We used these data to derive the Cambridge Multimorbidity Score, a single measure of multimorbidity for all adults, and the electronic frailty index (eFI). While the eFI can be used from the age of 50 years, we will use it in the coming season for people aged 65 years or older [52,53].

Our data are linked to national collection of hospital and death data, so we can report severe outcomes [54]. Our data are also linkable with the National Immunization Management System, which provides vaccination data for COVID-19 and influenza in England. These data will be used to estimate VE, with mid-season and end-of-season studies for influenza, an autumn VE study for COVID-19 vaccines, and a burden of disease study for RSV. These results will contribute to the Joint Committee on Vaccination and Immunization impact of vaccine policy, contribute to WHO reviews, and be published in peer-reviewed journals. 

The protocol for our VE studies is included in Multimedia Appendix 3.

### Virology and Serology Sampling and Testing

Ongoing recruitment and checking the representativeness of the network by region will ensure the RSC is representative. Maps of the distribution of virology and serology sampling practices and the entire network are included in Multimedia Appendix 4. We plan to increase the number of virology samples taken each week. The total so far for the 2022-2023 season is 11,001, with 878 as the highest number of virology swabs collected in week 51 of the year 2022. The median weekly total is 259 samples, with an interquartile range of 173 to 304 samples. These data have been and will be used to estimate VE for influenza and COVID-19 vaccines with a test-negative design, and can be used in the future to study the VE of RSV.

**Figure 6:**
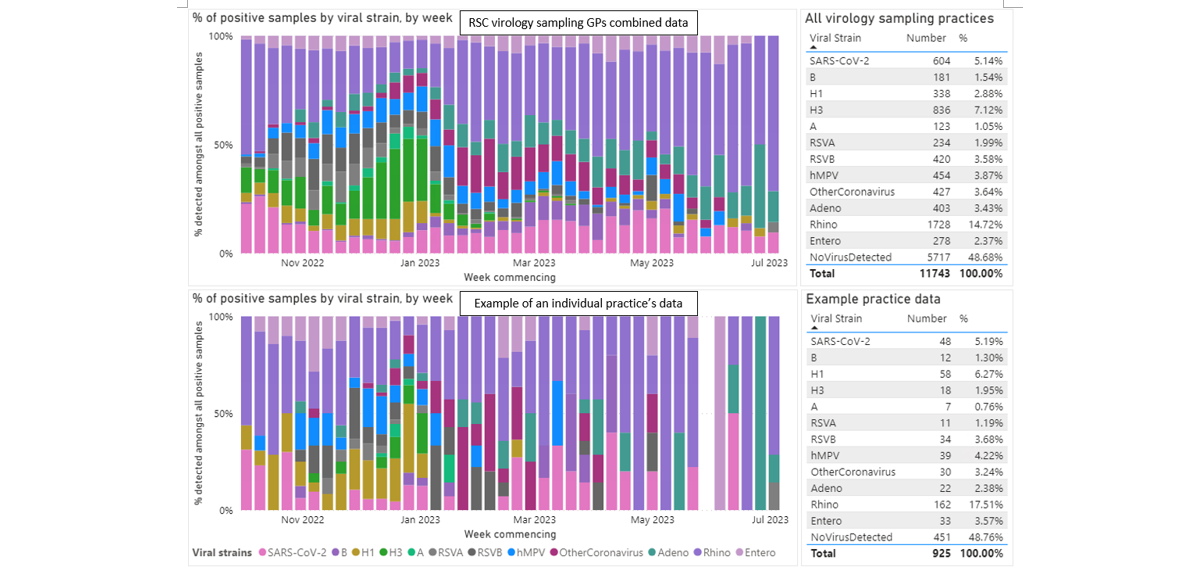
Circulating virology data taken from our observatory showing the percentage of positive samples by viral strain for Research and Surveillance Centre (RCS) general practitioners (GPs) combined and an example of an individual practice (2022-2023 season). hMPV: human metapneumovirus; RSV: respiratory syncytial virus.

Applying COM-B, our practice liaison team will be working with practices to achieve higher rates of virology samples. We will be continuing our regular visits and weekly and monthly reports to practices, and the scope will be driven by our learning from visits about how best to change (increase) sampling behavior and from our PPI input (Multimedia Appendix 5). Our virology dashboard provides practices with a comparison of what viruses are circulating in their practices compared with nationally (Figure 6). This can support practices to review their antibiotic prescriptions and antiviral medication uptake, and support better antibiotic stewardship.

**Figure 7:**
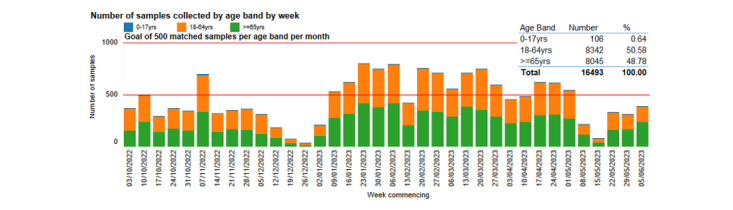
Serology data taken from our observatory and dashboard showing the number of samples collected by age band for Research and Surveillance Centre general practitioners combined (2022-2023 season).

We will need to control numbers in subgroups to achieve better representativeness of our serology sampling in the coming year, and produce a dashboard that practices can use to monitor activity (Figure 7). We will implement a technology-driven or manual approach to ensure national representativeness by age band and region. We will also offer pediatric phlebotomy training to encourage sampling in young people and children (Multimedia Appendix 6). The scale of serology sampling will be determined according to season.

### New Technologies and Capabilities

**Figure 8:**
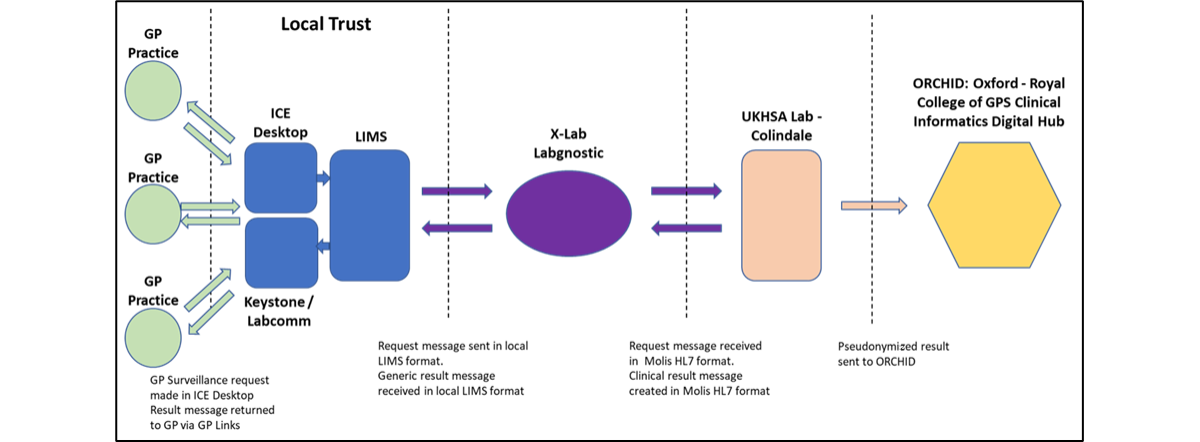
Virological surveillance data workflow. Samples are taken in practice. They pass through the local pathology laboratory to the UK Health Security Agency (UKHSA) viral reference laboratory at Colindale. Virology results go back to the general practitioner (GP) and patient and also into the Research and Surveillance Centre (RSC) database. ICE: Integrated Clinical Environment; LIMS: Laboratory Information Management System.

We plan to integrate LabLinks into the RSC. Currently, customized kits are provided to RSC practices for virology and serology sampling, and these kits are returned to UKHSA laboratories through the post. We plan instead to integrate RSC sampling with the electronic pathology test–requesting system currently integrated into primary care CMR systems. The details of the LabLinks program are described in Multimedia Appendix 7. Figure 8 provides an overview of the process.

**Figure 9:**
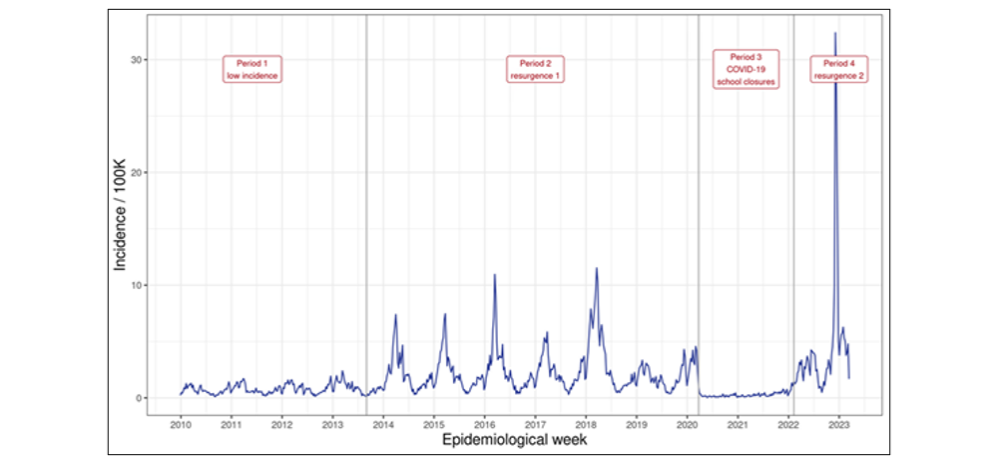
Weekly incidence of scarlet fever or streptococcal sore throat presenting to the English primary care sentinel network practices from 2010 to 2023. The incidence in the last quarter of 2022 was higher than that seen in the previous 10 years.

We will create a POCT nested cohort of practices willing to participate in feasibility studies. The key area of interest is POCT for group A streptococcal infection because there was a higher peak in the incidence of group A streptococcal infection in late 2022 than in the previous 8 years (Figure 9). A protocol for using molecular POCT is included in Multimedia Appendix 8 [55-57].

We will test EMIS Recruit [58] as a messaging system to invite targeted risk groups (immunocompromised) or younger people who had a booked blood test to consider volunteering to provide an extra blood sample for serology. The messaging system runs through EMIS Recruit to detect patients in target groups that have a recent blood test request. A message is sent to the patients to invite them to participate in our serology sampling. The patients will remind the practitioners of their eligibility at the blood test appointment. We will also be asking practices whether there would be interest in pediatric phlebotomy training to increase the sample number in younger children.

We will recruit one or two virology sampling practices per region that volunteer to collect urinary samples for UAG testing to infer pneumococcal infection.

We plan a pilot study of asymptomatic virology sampling. We will start with children under 5 years of age coming for vaccination. We may then move on to include people aged 40 to 74 years attending NHS health checks and people aged 40 years or older attending hypertension clinics in primary care.

### Creation of a Biomedical Resource

**Figure 10:**
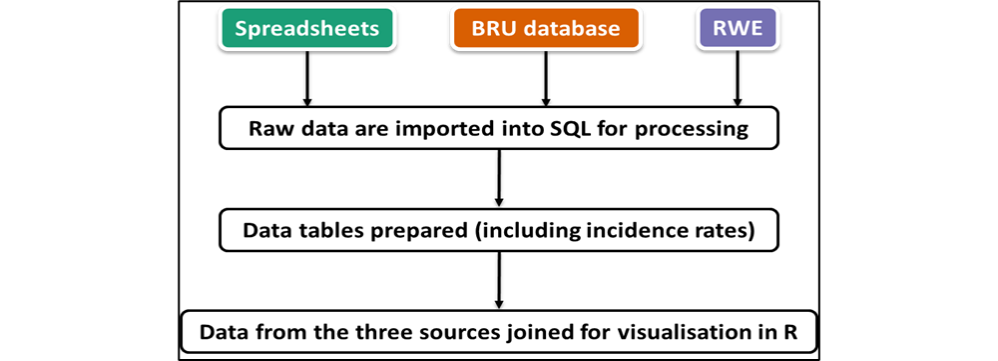
The assembly of data since 1967 within the biomedical resource. Birmingham Research Unit (BRU) was the location of the Research and Surveillance Centre up to 2013. Up to 1994, spreadsheets were loaded into an Access database, which was then replaced by structured query language (SQL). The Real World Evidence (RWE) server was set up in 2013.

**Figure 11:**
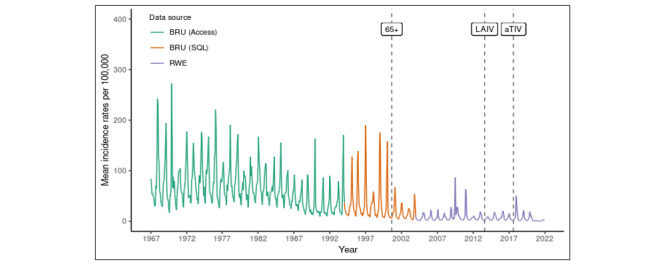
Change in the rate of presentation of patients with influenza-like illness to primary care from 1967 to 2022. The year of introduction of different flu vaccines has been added, and the data are 4 weekly averages. The point “65+” indicates the introduction of the flu vaccine for all people aged 65 years or older. “LAIV” indicates the introduction of the live attenuated influenza vaccine for school children. “aTIV” indicates the introduction of the adjuvanted trivalent influenza vaccine to improve vaccine effectiveness in people aged 65 years or older. BRU: Birmingham Research Unit; RWE: Real World Evidence; SQL: structured query language.

The RSC has unique longitudinal data stretching back to 1967. Currently, we are progressing with the assembly of these data into a single quinquagenarian resource (Figure 10). We have curated ILI incidences between 1967 and 2022 as an example (Figure 11).

### Legal Basis and Governance Framework for Conducting Sentinel Surveillance

Surveillance is authorized each year through a commissioning letter, authorized by the UKHSA, and sent out to all RSC sentinel network general practices. Privacy notices for individuals registered at a GP within the network are publicly available [59]. We request all member practices to share these with their registered patients.

### Evaluation of RSC Surveillance Compared With the WHO Mosaic Framework

The RSC meets many of the surveillance objectives of the WHO mosaic framework but within the scope of its virology plus primary and secondary care data (Table 1).

In Domain I (*detection and assessment of respiratory viruses*), we have a comprehensive virology panel and a nationally representative primary care network. Our data are strong but could be stronger with respect to having detailed information about clinical presentation. Our household key and information about residential care provide only limited information about transmission [43], and piloting whether there is asymptomatic spread will provide additional evidence about the spread of the disease.

In Domain II (*epidemiological characteristics of respiratory viruses*), our data are strong. Our linked data set allows us to monitor severe outcomes and mortality. We do not have sufficient coverage of all high-risk settings and do not collect data from hospitals where nosocomial infection is common. We can readily identify community-recorded vulnerable populations, and we term these “risk groups” [60]. We do not directly measure whether health care systems are overwhelmed, but we do record community rates of illnesses compared with other years [61].

In Domain III (*informing about health interventions*), we are able to infer the impact of interventions, such as lockdowns and shielding, during the COVID-19 pandemic, and we can see from our data the impact of school closures [62,63]. We are strong in measuring vaccine uptake and effectiveness, and our data have been used for vaccine adverse events [60,64]. We only have limited abilities to assess the effectiveness of some antivirals and other therapeutics owing to their central administration, and our capabilities are greater where these are recorded in the GP CMR. We have the capability to assess diagnostic tests and will compare POCT with reference virology laboratory results [55,65]. NHS England ARI hubs are planned to join the network, and we plan to evaluate the impact of these networks. We do not provide candidate vaccine viruses.

The RSC has additional surveillance objectives (Table 2). PPI and bacterial surveillance are essential. We could have a role at the system level of exploring how POCT might have an impact on treatment selection and health outcomes. Additionally, we see compliance with information governance standards as essential and linkage between clinical and viral sequencing data as enabling genomic surveillance.

**Table 1:** Research and Surveillance Centre implementation of the World Health Organization mosaic framework.

WHO^a^ domain and WHO surveillance objective			RSC^b^ delivery^c^		Completeness
**Domain I: Detection and assessment**					
	Rapidly detect outbreaks and other events	Our sentinel network covers over 32% (N=19 million) of the population and includes virology and serology sampling. A new ARI^d^ phenotype and POCT^e^ enhance the capability.		Full delivery: RSC and UKHSA^f^	
	Assess transmissibility and its risk factors, and extent of infection	RSC data include a household key to identify any household spread, and we can identify people in residential homes. We are starting asymptomatic testing.		Partial delivery: RSC and UKHSA	
	Describe clinical presentation and risk factors for severe outcomes	Our ARI phenotype includes, as child concepts, most clinical presentations. We have specified key clinical data to collect. Links to hospital data provide severe outcomes.		Full delivery: RSC and UKHSA	
**Domain II: Monitoring epidemiological characteristics**					
	Monitor characteristics of illnesses over time	Our surveillance of ILI^g^, ARI, and SARI^h^, applying the ARI phenotype, enables the ongoing monitoring of respiratory illnesses over time.		Full delivery: RSC and UKHSA	
	Monitor characteristics of circulating viruses	Our collaboration with the UKHSA in virology and serology sampling (including asymptomatic individuals) supports the monitoring of circulating viruses.		Full delivery: RSC and UKHSA	
	Monitor high-risk settings and vulnerable populations	Long-term investment in UK health computing and pay-for-performance means that primary care records capture risk groups. Other settings may be excluded.		Partial delivery: UKHSA from other settings	
	Monitor the impact on and coping abilities of health care systems	We can make year-on-year comparisons of data, running back over many years. However, there are no specific “coping abilities.”		Partial delivery: RSC and UKHSA	
**Domain III: Informing use of interventions**					
	Monitor the impact of nonmedical interventions	We have conducted epidemiological studies to explore the impact of nonmedical interventions during COVID-19 (eg, shielding).		Exemplar studies: RSC and UKHSA	
	Provide candidate vaccine viruses	We do not provide candidate vaccine viruses as part of surveillance.		Out of scope	
	Vaccine coverage, effectiveness, impact, and cost-effectiveness	Standardized national data indicate excellent coverage and impact. We have the capacity to supply data for vaccine effectiveness and cost-effectiveness studies.		Partial delivery: RSC and UKHSA	
	Monitor the effectiveness of antivirals and other therapeutics	We have conducted studies on the effectiveness of antivirals but have limited ability to assess new therapies owing to their central administration and data access issues.		Exemplar studies: RSC and UKHSA	
	Monitor the effectiveness of diagnostic tests	We have provided a comparison of results from POCT and UKHSA reference virology laboratories. This work could be scaled; see our additional objectives.		Exemplar studies: RSC and UKHSA	
	Monitor the effectiveness of clinical care pathways	We can monitor care pathways where we have access to data. Gaps include out-of-hours, NHS 111, and care homes. UKHSA syndromic surveillance fills these gaps.		Partial delivery: RSC and UKHSA	
	Monitor adverse events to vaccines and therapeutics	Ad hoc studies monitor adverse events of interest, either through data (passively) or by providing additional questionnaires. This is not a systematic part of surveillance.		Exemplar studies: RSC and UKHSA	

^a^WHO: World Health Organization.

^b^RSC: Research and Surveillance Centre.

^c^Each row is cumulative. Only new features are added in each row.

^d^ARI: acute respiratory infection.

^e^POCT: point-of-care testing.

^f^UKHSA: UK Health Security Agency.

^g^ILI: influenza-like illness.

^h^SARI: severe acute respiratory infection.

**Table 2:** Research and Surveillance Centre additional surveillance objectives that might be added to the World Health Organization mosaic framework.

Additional RSC^a^ objectives	RSC delivery^b^	Completeness
PPI^c^	PPI is fundamentally important. We need the support of patients and the public for what we do. It is hard for us to fulfil our information governance responsibilities regarding informing patients and the public without strong PPI work.	Partial delivery: RSC and UKHSA^d^
Monitor bacterial infections	Primary and secondary bacterial infections are significant contributors to the burden of respiratory disease. Informing hospitals about these pressures is challenging without considering bacterial diseases. Our innovations include (1) POCT^e^ for group A streptococcal infection and (2) urinary antigen tests to test for pneumococcus infection. We plan to improve the use of routinely collected bacteriology data.	Piloting by the RSC in collaboration with the UKHSA
POCT system-level evaluation	While we have evaluated POCT at the individual test level (mainly the sensitivity and specificity of specific virological tests), the RSC has the capability to evaluate the effectiveness of POCT at the system level. Outcomes could include: (1) antimicrobial stewardship, (2) use and effectiveness of antivirals, and (3) early intervention in high-risk individuals or populations.	Piloting by the RSC
Secured data governance	Our data governance portfolio, including the commissioning letter as described in the legal basis and governance framework for conducting sentinel surveillance, facilitates secure data use within the RSC.	Legal and national standards met by the RSC
Genomic surveillance resource	Our biomedical resource will link clinical data to virology sequencing data held in a publicly accessible resource. This will enable the linkage of clinical disease features and outcomes to the viral genomic sequence.	Partial delivery: RSC and UKHSA to complete this Wellcome Trust–funded project in 2024

^a^RSC: Research and Surveillance Centre.

^b^Each row is cumulative. Only new features are added in each row.

^c^PPI: patient and public involvement.

^d^UKHSA: UK Health Security Agency.

^e^POCT: point-of-care testing.

## Discussion

### Principal Findings

The RSC and UKHSA are providing the most comprehensive primary care respiratory infection surveillance in the United Kingdom. We have extended the number of surveillance approaches for the coming season, meeting more of the areas proposed in the WHO mosaic framework (Table 1) [19].

In Domain I (*detection and assessment of respiratory viruses*), we have a robust system that has run over decades. However, we have scope to improve the rapidity and reliability of our results by increasing our sampling numbers and data quality so we can better detect changes.

In Domain II (*epidemiological characteristics*), we also have a robust system. There is potential to integrate work about asymptomatic infection, do more to include high-risk settings in our system, and develop indicators of our health care system’s ability to cope.

Domain III (*informing the use of health interventions*) is an area where we provide some key data, but this could be strengthened. We are able to monitor the impact of nonmedical interventions like social distancing [62,63], but other data are also needed to extend our capability of informing health interventions. We do report vaccine coverage, though data about vaccine exposure where vaccines are not given in primary care are more limited. We have primary care data and can link to other data to monitor the effectiveness of antivirals and other therapies, but access to centrally held data sets can be slow. We are well placed to report VE and adverse events of interest following vaccination [37,54] rapidly when using primary care data but with a greater lead time when we need to link to secondary care data. We can measure the costs of medically attended conditions. However, including measures of health-related quality of life would extend our ability to assess the disease burden.

Finally, we propose additional objectives that might be added (see Table 2). Among these, we consider that PPI and addressing the burden of primary and secondary bacterial infections are the most important.

### Comparison With Prior Work

The strength of the RSC’s sentinel surveillance has long been established, but it has now been greatly extended, with the network more than doubling in size during the pandemic [66]. Other countries adapted their surveillance during the COVID-19 pandemic, including setting patient sampling routes outside of primary care (Sweden, Netherlands, and Scotland), decentralizing reference testing laboratories (France, Portugal, Scotland, and Spain), and optimizing digital data collection (Sweden, Netherlands, England, Scotland, France, Portugal, and Spain) [67]. Most of these changes were temporary, but the changes in the RSC network, such as the expanded sentinel practices and the introduction of electronic links to laboratories, have remained and will be further developed.

Australia, Belgium, the Netherlands, and the United States have shown that GPs would like to use more POCT to help them diagnose acute conditions [68]. Currently, the Welsh government has introduced a pharmacy-led service to undertake a structured clinical assessment using clinical prediction scores and POCT for cases of suspected strep A infection [69].

### Strengths and Limitations

The strengths of our network are its size (just under a third of the English national population), the level of sampling, and the commitment to improving data quality. The network has shown adaptability through the COVID-19 pandemic and a strong partnership working with the UKHSA. We also have collaborations with other European sentinel networks and international collaborations [67]. The United Kingdom has a registration-based system that is free at the point of care, which allows good population coverage and facilitates the presentation of population-based infection rates. A unique national ID, the NHS number, ensures that primary care data can be linked to hospital and death data, allowing severe outcomes to be reported.

The limitations of routine data are that they are recorded by busy clinicians often working under pressure, and thus, they can be incomplete. Despite our best efforts, there can be gaps in data quality [70]. It is inevitable that our data will not capture all cases, and disease etiology might not be precise. While primary care data can be reliably reported within 3 days in arrears, it is much slower to gain access to secondary care data and other data sets. The national policy is to move toward a smaller number of secure data environments, and the RSC may need to migrate into one of these [21]. NHS primary care is increasingly working at scale, with the NHS setting up ARI hubs to work across geographical areas. We are exploring recruiting ARI hubs into our network.

### Conclusions

The RSC has grown and adapted through the pandemic. Our biggest areas of change will be the introduction of an ARI phenotype, using technology to reduce the barriers to virology sampling and hopefully increase the scale and representativeness of virology sampling. The challenges in implementing change and the requests for more consistent data recording risk discouraging practices from remaining part of the RSC. Overall, our plans for the coming season will deliver more of the WHO surveillance mosaic.

## Appendix

Multimedia Appendix 1 Patient and public involvement: Images from the first monthly newsletter.

Multimedia Appendix 2 Coding virology swab results.

Multimedia Appendix 3 Proposals for vaccine effectiveness studies for the coming season.

Multimedia Appendix 4 Distribution of virology and serology sampling practices and the entire network.

Multimedia Appendix 5 Practice liaison team communications.

Multimedia Appendix 6 Summary of a pilot study to introduce pediatric phlebotomy training in the Research and Surveillance Centre network by region.

Multimedia Appendix 7 An introduction to LabLinks.

Multimedia Appendix 8 A protocol for a study using point-of-care testing.

## Abbreviations

ARI: acute respiratory infection
CMR: computerized medical record
eFI: electronic frailty index
FHIR: Fast Healthcare Interoperability Resources
GISRS: Global Influenza Surveillance and Response System
GP: general practitioner
hMPV: human metapneumovirus
ILI: influenza-like illness
LRTI: lower respiratory tract infection
NHS: National Health Service
PhEMA: Phenotype Execution Modeling Architecture
POCT: point-of-care testing
PPI: patient and public involvement
RCGP: Royal College of General Practitioners
RSC: Research and Surveillance Centre
RSV: respiratory syncytial virus
SARI: severe acute respiratory infection
SNOMED CT: Systematized Nomenclature of Medicine Clinical Terms
UAG: urinary antigen
UKHSA: UK Health Security Agency
URTI: upper respiratory tract infection
VE: vaccine effectiveness
WHO: World Health Organization
