# Supplementary material for: Postpandemic Sentinel Surveillance of Respiratory Diseases in the Context of the World Health Organization Mosaic Framework: Protocol for a Development and Evaluation Study Involving the English Primary Care Network 2023-2024
Source: JMIR Public Health Surveill. 2024 Apr 3;10:e52047. doi: 10.2196/52047 (PMC11024753; doi:10.2196/52047)

**Multimedia Appendix 1.** Patient and public involvement: Images from the first monthly newsletter.


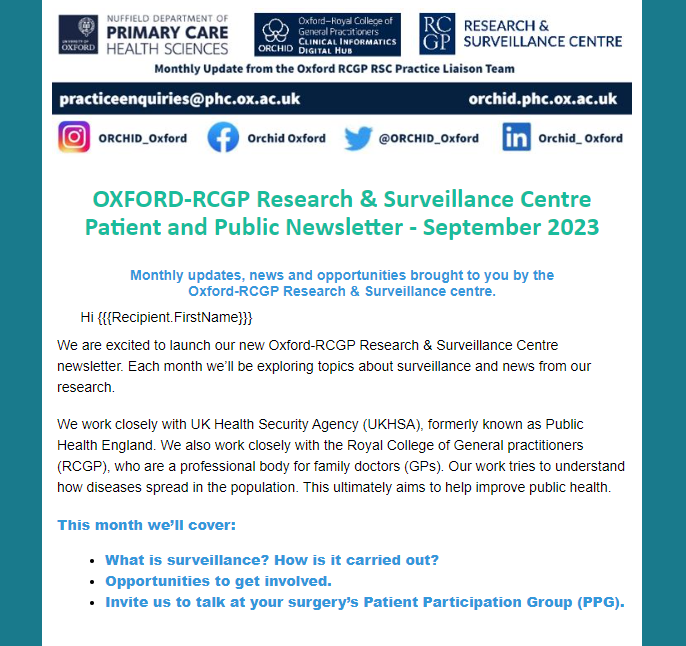


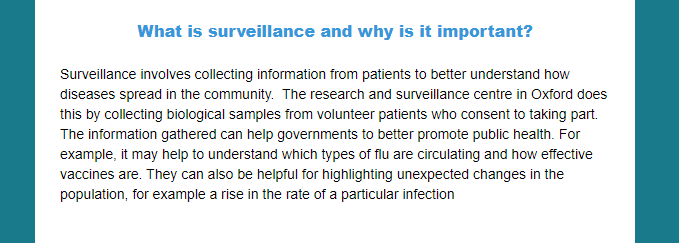


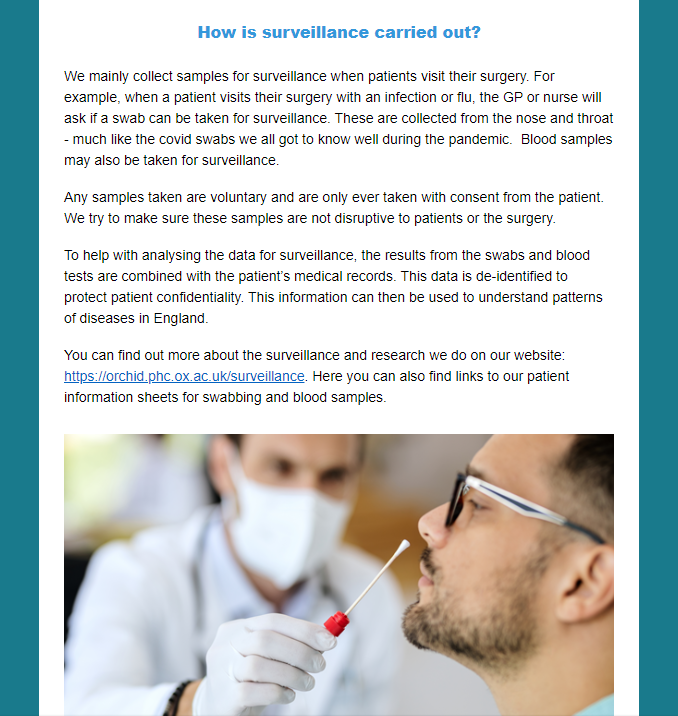


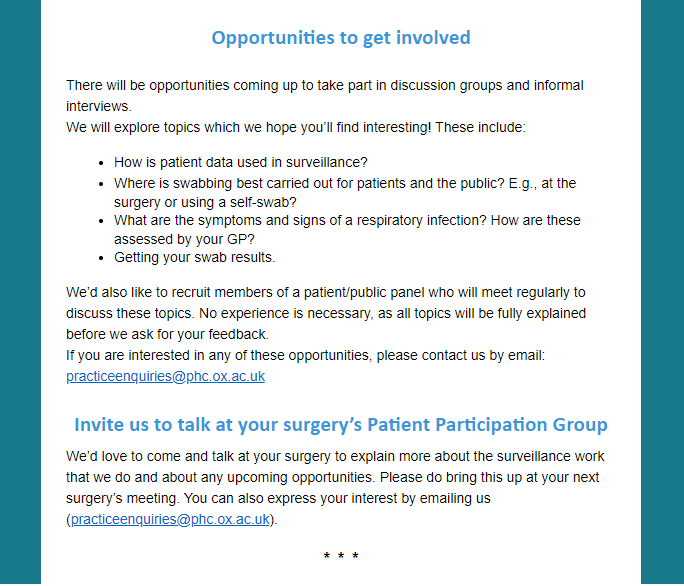

Supplement: Multimedia Appendix 1 [file publichealth_v10i1e52047_app1.docx]
