# Supplementary material for: Postpandemic Sentinel Surveillance of Respiratory Diseases in the Context of the World Health Organization Mosaic Framework: Protocol for a Development and Evaluation Study Involving the English Primary Care Network 2023-2024
Source: JMIR Public Health Surveill. 2024 Apr 3;10:e52047. doi: 10.2196/52047 (PMC11024753; doi:10.2196/52047)
Supplement: Multimedia Appendix 2 [file publichealth_v10i1e52047_app2.docx]

# Multimedia Appendix 2. Coding virology swab results.

Please enter the code with the attached date of the day that the swab was taken, not when the result was received.

There are some codes that are available in SystmOne but not in EMIS.

**Seasonal Coronavirus**

| **Term** | **SNOMED CT code** | **Read code** |
| --- | --- | --- |
| *Coronavirus Infection* | 186747009 | A795 |

**Pandemic Coronavirus – COVID-19 or SarS-CoV-2**

| **Term** | **SNOMED CT code** | **Read code** |
| --- | --- | --- |
| *Severe acute respiratory syndrome coronavirus 2 ribonucleic acid detected* | 1324601000000106 | Y2a3b |

There are no codes for the individual genes listed on the UKHSA results.

**Seasonal Influenza**

Please code the subtype if that result is available.

| **Term** | **SNOMED CT code** | **Read code** |
| --- | --- | --- |
| *Influenza A virus subtype H1 present* | 441043003 | XaPIN |
| *Influenza A virus subtype H3 present* | 441049004 | XaPIP |

If there is no subtype specified use the code below and add “positive”

| **Term** | **SNOMED CT code** | **Read code** |
| --- | --- | --- |
| *Influenza A virus ribonucleic acid detection assay* | 1008261000000108 | XabpY |

**Respiratory Syncytial Virus – RSV**

| **Term** | **SNOMED CT code** | **Read code** |
| --- | --- | --- |
| *Respiratory syncytial virus untyped strain present* | 441278007 | XaPOc |

**Human Metapneumovirus**

| **Term** | **SNOMED CT code** | **Read code** |
| --- | --- | --- |
| *Human metapneumovirus present* | 441133003 | XaPOd |

**Adenovirus**

| **Term** | **SNOMED CT code** | **Read code** |
| --- | --- | --- |
| *Human Adenovirus Present* | 440930009 | XaPOI |

**No Virus Found**

We don’t check for every virus or any bacteria so some of these are inevitable, even if infection is present.

| **Term** | **SNOMED CT code** | **Read code** |
| --- | --- | --- |
| *No respiratory virus detected* | 365791000000102 | XaPOq |
