# Supplementary material for: Postpandemic Sentinel Surveillance of Respiratory Diseases in the Context of the World Health Organization Mosaic Framework: Protocol for a Development and Evaluation Study Involving the English Primary Care Network 2023-2024
Source: JMIR Public Health Surveill. 2024 Apr 3;10:e52047. doi: 10.2196/52047 (PMC11024753; doi:10.2196/52047)
Supplement: Multimedia Appendix 3 [file publichealth_v10i1e52047_app3.docx]

# Multimedia Appendix 3. Proposals for vaccine effectiveness studies for the coming season.

**Study 1: The effectiveness and impact of the national COVID-19 immunisation programme using the sentinel practice network and database in England: Autumn 2022 through Spring 2024 Proposal**

**1. Background**

SARS-CoV-2 vaccination was introduced in the UK on 7th December 2020. Initial clinical trial results and population studies on vaccine effectiveness (VE) showed that the vaccines were successful in preventing infection, but population studies suggest that this has lessened with antigenic change in the virus over time, though effectiveness against severe outcomes remains moderate. Continued monitoring in the wider population is important to optimize vaccination strategy to minimize the burden of disease. A large GP data source will help detect differences in VE between clinical groups.

Strong systems to evaluate the effectiveness of the COVID-19 vaccine programme will enable to:

- optimise recommendations for the use of the vaccine
- target complementary or alternative public health measures (e.g. antivirals) for population segments where vaccine is less effective.
- allow more precise estimates of the impact of current vaccination strategies on the burden of disease to support the national vaccination campaign
- evaluate COVID-19 vaccines to better understand optimal composition; use of adjuvants; need for booster doses.
- better manage and respond to reports of vaccine failures.
- counterbalance the reports of adverse events following immunisation by providing elements for an adequate risk management and cost-effectiveness analysis.

This proposal concerns the use of the established general practice database maintained by the Oxford-Royal College of General Practitioners (RCGP) Research and Surveillance Centre (RSC), one of Europe’s oldest sentinel systems.^[[1]](#footnote-1)^ The RCGP RSC has supported and collaborated with Public Health England and predecessor bodies for over 50 years and adapted significantly to meet the challenges of the pandemic.^[[2]](#footnote-2),^^[[3]](#footnote-3)^

We will use cohort and case-control methods to estimate COVID-19 VE against a range of clinical and virological endpoints, with a focus on the 2022 Autumn, 2023 Spring and 2023 Autumn booster programmes.

We propose the dataset for analysis aims to include:

1] The inclusion of results of laboratory tests via both Pillar testing and Sentinel surveillance, both historical and current.

2] Information on the type and dose of vaccine given, manufacturer and batch number.

3] Patient specific information on demographic variables, risk group status and prior influenza vaccine history.

4] Further linked relevant outcome data, in particular hospital admission and mortality data.

**2. Objectives**

**2.1 Aim**

For the 2023/24 period, the aims are to:

- Describe the population that have received a booster vaccine at the time of data extraction.
- Estimate COVID-19 vaccine effectiveness for a range of clinical and virologically confirmed end-points in primary care across all age groups.

**2.2 Primary objectives**

The key questions to be addressed for confirmed COVID-19 infection and clinical outcomes in 2023/24 in relation to vaccine effectiveness, taking into account previous vaccination and booster vaccination, will be to estimate:

- Overall VE by vaccine type (mRNA or other)
- VE by specific clinical risk group
- VE by time since vaccination
- VE by time period, depending on the timing of boosters and circulation of specific variants

| Outcomes combine confirmed and clinical elements:  **Confirmed**: Swab either taken   - via GP from persons presenting with COVID-like symptoms. Laboratory investigation undertaken in the PHE Respiratory Virus Reference laboratory to detect positive swab for SARS-CoV-2 and other respiratory viruses.   **Clinical:**   - COVID-19 coded hospitalisation - COVID-19 coded death - death within 28 days of positive SARS-CoV-2 test. |
| --- |

**2.3 Secondary objectives**

Descriptive statistics

To describe Spring and Autumn 2023 booster vaccine uptake in the RCGP population by target groups and by demographic and clinical variables.

**3. Methods**

**3.1 Study designs**

For 2023/24 RCGP RSC and UKHSA in collaboration will use two main study designs to estimate COVID-19 vaccine effectiveness in England across all age groups, and in sub-populations for individuals registered with participating GPs in the RCGP RSC sentinel network. The primary designs to be used are as follows:

- Cohort study with primary outcomes hospitalisation and death.
- Test-negative design (TND) study on patients within the sentinel swabbing scheme.

This proposal covers both the cohort study and the TND study.

**3.2 Study population and data source**

| Study population to be included:  Cohort study: Approx. 18,800,000 registered persons in England. About 20% of the study population are ≥65 years.  Swab based TND study: Restricting to those consulting their GP with symptoms and swabbed via the sentinel scheme, available positive cases and negative controls will depend on current prevalence and swab rates.  Spring 2023 boosters, will restrict to: individuals aged 75+, immunosuppressed individuals aged 16+  Autumn 2022/2023 boosters, will restrict to: individuals aged 50-74, individuals aged 75+, individuals aged 16+ in clinical risk groups |
| --- |

**3.2.1 Cohort study population**

***Definition***

The cohort study population is composed of the entire registered population in the participating practices in England. This will be those registered on or before the start of vaccine booster rollout.

The data capture methods include the collection of all relevant potential confounding variables (e.g. variables that may be associated with eligibility for vaccination and the outcome of interest) and information on vaccine type.

**Exclusion criteria**

Registered patients will be excluded from the data extract if they have expressed a wish to be excluded from the surveillance programme.

Logical checks will be performed and patients identified with nonsensical records will be excluded. Those with unusual COVID-19 vaccine histories will be excluded.

**GP computerised databases**

Individual-level data is extracted twice-weekly from GP computer systems into the RCGP RSC database. SNOMED clinical terms^[[4]](#footnote-4)^ are used to identify outcomes, risk factors, cases of COVID-19 and vaccination events.^[[5]](#footnote-5)^ Routines to extract and manipulate the data into the correct format for statistical analysis are required.

**3.2.2 TND study population**

**Definition**

The TND study population is composed of individuals that have contented to a respiratory swab as part of the sentinel swabbing scheme.

**Exclusion criteria**

- Logical checks will be performed and patients identified with nonsensical records will be excluded.
- Those with unusual COVID-19 vaccine histories (vaccination outside the campaign period or receiving an alternative vaccine to the main offer – see section 3.5).
- SARS-CoV-2 negative controls with a positive influenza test will be excluded for analyses that span the main period of influenza circulation (i.e. Autumn booster evaluations, but not Spring booster evaluations).
- Patients swabbed >10 days post onset.
- Patients whose swab date cannot be determined.
- Patients with no linked vaccination history or clinical risk status.

**3.3 Study setting**

| **Cohort**  Total number of practices: ~1800,  Size of population: ~18,800,000 registered persons (all patients registered in the practices).  Representativeness: The population is closely representative of the English national population by region and by age and gender. The population is about one third of the national population.  For practical purposes this is a complete database of information collected in the sentinel networks. Given the programme of enhanced data extraction which is underway we would not expect to lose more than 2% of relevant data.  The practice data bases are an accumulated record of person-specific health-related data. We extract selected data to provide our routine surveillance program. |
| --- |

| ***TND***  Participants in the RCGP/UKHSA sentinel swabbing scheme. These are patients who contacted their GP, where the GP was part of the Royal College of General Practioners Research and Surveillance Centre (RCGP-RSC) sentinel scheme, and who had a sentinel respiratory swab taken with a result for SARS-CoV-2. >200 practices contribute sentinel swabs for virology.  Indications for swabbing are influenza-like illness, COVID19-like illness, an acute respiratory infection or exacerbation of asthma/COPD with onset within the last 10 days.  Patients are either swabbed and consented by their GP as part of a face-to-face consultation, or directed to take a postal self-swab by GPs through takeatestuk.com after being issued a voucher code.  All sentinel swabs are tested for respiratory infections including influenza and RSV using RT-PCR at UKHSA’s Virus Reference Laboratory. Vaccination data are available via linkage to GP records. |
| --- |

**3.4 Study period**

This proposal relates to an ongoing programme of disease surveillance. Data are collected continuously on a daily basis in England. It includes the diagnoses or problems reported at every consultation. The database can be interrogated in relation to any specific disease or in any defined period. It is assumed that the period of COVID-19 activity will continue throughout 2023 and 2024, this will be monitored through surveillance.

Autumn 2022 booster vaccine doses were offered to all age 50+ plus all members of clinical risk groups age 5+ and health and social care workers, carers or close contacts of the immunosuppressed. Autumn booster doses primarily included bivalent Original/Omicron BA.1 mRNA vaccines (Pfizer-BioNTech, Comirnaty or Moderna, Spikevax). The autumn booster vaccination campaign opened 5 September 2022 and closed 12 February 2023.

Spring 2023 boosters were offered to those age 75+ or those with weakened immune systems, during the period 3 April – 30 June 2023. Vaccines given were primarily either bivalent Original/Omicron BA.4-5 mRNA vaccines (Pfizer-BioNTech, Comirnaty) or monovalent Beta recombinant vaccines (Sanofi-GSK, VidPrevTyn).

From Autumn 2023, a new booster vaccine dose will be offered to all age 50+ plus all members of clinical risk groups age 5+ and health and social care workers, carers or close contacts of the immunosuppressed. Autumn booster doses will include monovalent XBB mRNA vaccines, bivalent Original/Omicron BA.4-5 mRNA vaccines, and monovalent Beta recombinant vaccines, depending on age and availability. The autumn booster vaccination campaign will be ongoing from September 2023.

The data may be examined in any arbitrary period according to interest. At the time of writing, Omicron XBB lineages are dominant, but this could change at any time, and variant may impact vaccine effectiveness. Study periods may be amended to cover variant-dominant periods of infection or according to decision makers needs. Cohort analyses may require censoring at the last date in records where outcomes appear to be well captured.

To evaluate the Autumn 2022 boosters, analyses will commence Monday 5 September 2022 and end Sunday 2 April 2023. We note that dominance of variants shifted from Omicron BA.5 at the beginning of this period, to Omicron BQ.1 from w/c 7 November 2022, to co-circulating Omicron XBB, XBB.1.5 and CH.1.1 from w/c 23 January 2023 with XBB.1.5 prevailing from w/c 20 February 2023, hence secondary analyses will explore splitting the study period accordingly.

To evaluate the 2023 Spring boosters, analyses will start on the Monday 3 April and end Sunday 3 September. Evaluation will occur September 2023, or as soon as linked data are available. Omicron XBB.1.5 was the dominant variant at the beginning of the study period.

To evaluate the 2023 Autumn boosters, cohort and TND analyses will start on the Monday of the first week of the booster programme. The TND analyses will be in conjunction with influenza VE, in January and May 2024. Evaluation via the cohort will be after the end of the influenza season.

**3.5 Exposure**

**Definition**

All persons will be considered fully booster-vaccinated 14-days after a booster dose.

**Ascertainment**

Vaccination status, date, manufacturer and type is extracted from the study database (and linked from other sources if necessary).

Individuals will contribute pre-booster person time up to the point of booster vaccination and thereafter assigned to categories based on combinations of time since dose and booster vaccination type

Time since dose:

- pre-booster/unboosted (baseline)
- 0-13 days,
- 14-91 days,
- 92-182 days,
- 183+ days

Type of vaccine:

- mRNA XBB
- mRNA bivalent original/BA.4-5
- recombinant Beta

These categories may be assigned separate factors for different manufacturers, as required.

The following are considered ‘odd’ vaccination histories, to be considered for exclusion:

- Other vaccine types than those listed in section 3.4 above
- Vaccination between 13 February and 3 April 2023 (for Spring 23 booster analysis)
- Vaccination between 31 June and 3 Sept 2023 (for Autumn 23 booster analysis)

**3.6 Outcome(s)**

**Outcomes for Cohort Studies of Severe Disease**

Linkage with ONS mortality data, Hospital Episode Statistics (HES) and/or Secondary Uses Service (SUS), should provide the following additional outcomes:

- A COVID-19 hospitalisation will be defined as hospital admission, with
  - a primary COVID-19 discharge code present. (ICD10 U07.1, U07.2, U07.4 or U07.5)
  - a primary acute respiratory code (J04, J09-J22, J80), in conjunction with BOTH a secondary COVID-19 discharge code AND a record of a positive SARS-CoV-2 test -14 to +2 days around admission (to ensure infection prior to hospitalisation)
  - consecutive admissions with the same primary code will be aggregated and the primary hospitalisations analysis will include only stays of 2 nights or longer
- Stays of any length will be evaluated in a secondary analysis
- A very severe hospitalisation definition including use of Oxygen or admission to intensive care in addition to the primary definition above will also be evaluated in a secondary analysis.
- A COVID-19 death will be defined as a death with a COVID-19 ICD10 code on the death certificate (in any position)
- A definition including additional deaths occurring within 28 days of a positive test will also be used in a secondary analysis.

Negative controls do not need to be identified.

**Outcomes for TND study of symptomatic GP visit**

TND requires both confirmed test positive cases and test negative controls to be identified. Both positive cases and negative controls should present to their GP with a defined set of symptoms, consistent with those required for participation in the sentinel swabbing scheme. Swabbing should be undertaken within ten days of onset of symptoms. Indications for swabbing include influenza-like illness, COVID19-like illness, acute respiratory infection, and asthma/COPD exacerbations.

A case is defined as an individual with a positive SARS-CoV-2 test via the sentinel swabbing scheme, with symptoms/illness within 10 days of onset.

A control is defined as an individual with a negative SARS-CoV-2 test via the sentinel swabbing scheme, with symptoms/illness within 10 days of onset AND (Autumn boosters only) influenza negative.

Multiple results may be available within any one individual, but a 90-day window between consecutive positive tests will be included in either study to ensure independence of positive outcomes.

Once timing of positive events have been established for the TND, the first negative result per 3 week period will be included, if at least 21 days before or 90 days after any positive result.

Analyses will initially exclude individuals presenting with asthma/COPD exacerbation, but indications for swabbing will be further explored in sensitivity analyses.

**3.7 Sub-groups**

Study subjects may be categorised according to age groups and Department of Health defined risk categories for COVID-19 vaccination.^[[6]](#footnote-6)^ The clinically high-risk groups include:

- chronic respiratory disease, including chronic obstructive pulmonary disease (COPD) and cystic fibrosis
- severe asthma
- chronic heart disease (and vascular disease)
- chronic kidney disease
- chronic liver disease
- chronic neurological disease including epilepsy
- diabetes
- immunosuppression due to disease or treatment, including solid organ, bone marrow and stem cell transplant recipients and people with specific cancers
- asplenia and splenic dysfunction
- morbid obesity
- severe and profound learning disability
- severe mental illness

Some clinical risk groups may be suitable to break down further, if required:

- chronic kidney disease, by severity
  - latest recorded stage 3 and latest GFR ≥ 30 and no record of dialysis
  - latest recorded stage 4-5 or latest GFR < 30 or record of dialysis
- diabetes, by type
  - type 1
  - type 2
- immunosuppressed
  - Conditions that compromise bone marrow function
  - Solid organ transplants
  - Immunosuppressive treatments
  - Cancer treatments
  - Primary and acquired immunodeficiencies

Definition and identification of risk groups:

| High risk is determined by age group and the presence of recognised risk morbidities recorded in the electronic health record for the patient concerned.  Pre-defined SNOMED codes have been established for these various underlying risk factors (rather than by questionnaire). We are in the process of establishing a formal agreement with Nottingham University PRIMIS to use the SNOMED CT codes that they have developed for these various underlying risk factors. In return for using these we have been asked simply to acknowledge the work done by Nottingham University in developing these clinical terms lists . |
| --- |

**3.8 Confounding factors and effect modifiers**

To control for differences in health status in vaccinated individuals compared with non-vaccinated, information on potential confounding factors is collected.

***Presence of chronic disease*** (according to Nottingham University PRIMIS specification)

- Major chronic disease groups identified in Section 3.7

**Propensity to consult**

- Number of consultations per patient for outcomes in previous year (e.g. Sep 2021-Aug 2022)
- Will be used to create age-standardised quartiles of consultation and a non-consulters group

**Indicators of co-morbidity and underlying ill health**

- Cambridge Mortality Measure (in age-standardised quartiles/quintiles).
- Smoking.
- Electronic Frailty Index

**Demographic**

- Age (on 31st August 2022, in 5 year bands up to age 89, then 90+),
- Sex,
- Ethnicity (in 5 broad groups)

**Deprivation**

- At patient level the standard English Index of Multiple Deprivation (IMD 2019) will be used in quintiles,
- at practice level if unknown at patient level.

**Location (Practice/ region)**

- If possible a factor for practice will be included in the analysis. This may prove difficult analytically, in which case NHS region will be used.

**Time/circulation**

- Week, using smoothing splines.
- Interaction with week and NHS regions, and week and age groups will be checked and the model with the lowest AIC (Akaikes’ information criterion)

**Influenza vaccination**

**Resident in a large household**

- Via household key. Single occupancy and large communal establishments have consistently been associated with test positivity and risk of mortality.^[[7]](#footnote-7)^,^[[8]](#footnote-8)^,^[[9]](#footnote-9)^
- We assign to groups based on median age <70, 70+ (e.g. care homes).

**3.9 Data Collection**

Data are collected by the GPs in their GP practice. They are extracted on a daily basis and made available to the RCGP RSC.

RCGP RSC will make a fully anonymised data extract available to UKHSA. Each individual will be given a unique identifier that can be used to link with serology and virology data.

RCGP RSC will separately share a hashed NHS number and the unique identifier for the purposes of linking to SGSS SARS-CoV-2 positive tests, and SUS / HES hospitalisation data held by UKHSA, along with the hashing key. Once extracted, the NHS number will be deleted and data retained with the unique identifier alone to link with the anonymised GP data records.

**Table 1: Data sources for each collected variable**

| Group of Variables | Variables | Data source |
| --- | --- | --- |
| Demographic characteristics | Age on 31 August 2022 | Electronic GP record |
|  | Gender | Electronic GP record |
|  | GP practice ID | Electronic GP record |
|  | Location – NHS region | Electronic GP record |
|  | IMD quintile | Electronic GP record |
|  | Ethnicity | Electronic GP record |
| Exposure | COVID-19 vaccinations | Electronic GP record / linkage to NIMS |
|  | Dates of vaccinations | Electronic GP record / linkage to NIMS |
|  | Type of vaccine / manufacturer | Electronic GP record / linkage to NIMS |
|  | Batch numbers | Electronic GP record / linkage to NIMS |
| Outcomes TND only | Laboratory confirmed outcome | Linkage to PHE virus reference lab data |
|  | Date of swab | Linkage to PHE virus reference lab data |
|  | Date of onset | Linkage to PHE virus reference lab data |
|  | Date of receipt | Linkage to PHE virus reference lab data |
|  | Presence of fever ±10d | Electronic GP record |
|  | Presence of cough ±10d | Electronic GP record |
|  | Presence of shortness of breath ±10d | Electronic GP record |
|  | Consultation for ILI ±10d | Electronic GP record |
|  | Consultation for COVID19 ±10d | Electronic GP record |
|  | Consultation for URTI ±10d | Electronic GP record |
|  | Consultation for LRTI ±10d | Electronic GP record |
|  | Consultation for ARI ±10d | Electronic GP record |
|  | Consultation for Bronchitis ±10d | Electronic GP record |
|  | Consultation for Bronchiolitis ±10d | Electronic GP record |
|  | Consultation for asthma/COPD ±10d | Electronic GP record |
| Outcomes cohort only | Date of hospital admission | Linkage to HES / SUS data |
|  | Date of discharge | Linkage to HES / SUS data |
|  | List of ICD10 discharge codes | Linkage to HES / SUS data |
|  | Use of Oxygen | Linkage to HES / SUS data |
|  | Date of ICU admission | Linkage to HES / SUS data |
|  | Date of Death | Electronic GP record / linkage to ONS mortality data |
|  | Date of positive SARS-CoV-2 test | Linkage to SGSS / PHE virus reference lab data |
| Confounding factors | Smoking status | Electronic GP record |
|  | List of PRIMIS risk groups | Electronic GP record |
|  | Electronic Frailty Index | Electronic GP record |
|  | Indicator of living in a large residence | Electronic GP record (household key) |
|  | Cambridge Mortality Measure | Electronic GP record |
|  | Consultation count | Electronic GP record |
|  | Influenza vaccination | Electronic GP record |

The absence of any of those characteristics in the database is assumed to indicate no presence of the characteristic under study (missing values are coded absent).

Covariate values are updated on 1^st^ September of each study year on information recorded in the study database.

| Continuous update of co-variates takes place. However, some need to be defined pre-season for analysis. |
| --- |

**Procedures for database management**

| Who enters data? GPs (E), GPs and authorised practice staff (S)  Who validates data? Not validated (other than by consistency in regular analyses)  Who links database? UKHSA links to hospital (SUS/HES) and testing (SGSS) data  How are data extracted? Automated data extraction according to agreed protocol  Who centralises data? University of Oxford acts as Trusted Research Environment (TRE) for these data.  Who analyses data? UKHSA, with input from RCGP RSC  Software used? Multiple systems. The Oxford-RCGP RSC database operates in an NHS Digital – Data Security and Protection (DSP) compliant TRE. Data held in this network are stored using SQL, with extracts to fit with any required output. |
| --- |

**3.10 Sample size**

This has been assessed using the calculator below, assuming 80% power and 5% significance level.

<https://apps.p-95.com/drivesamplesize/>

**Cohort**

The plots below shows the bottom end of the 95% CI for an attack rate of 1% over the study period for sample sizes from 10,000 to 1,000,000 (note that the size of the RCGP RSC population in the smallest risk group is over 60,000). Attack rate is based on a rate of 15 cases per 10,000 population (~Nov 2020) over a 6-7 week period.

A : 60% VE


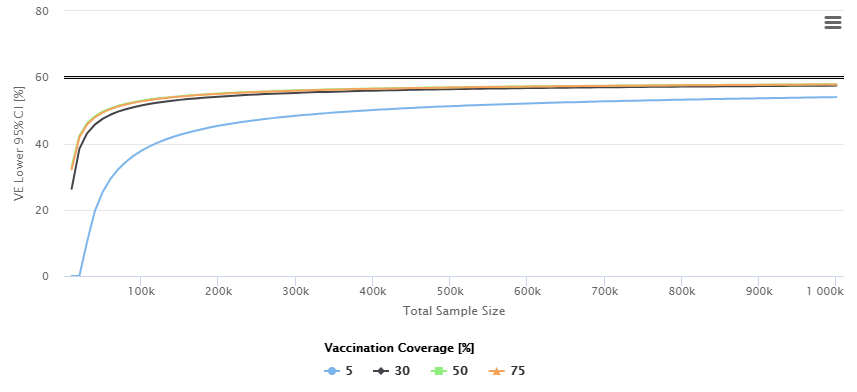


B : 90% VE


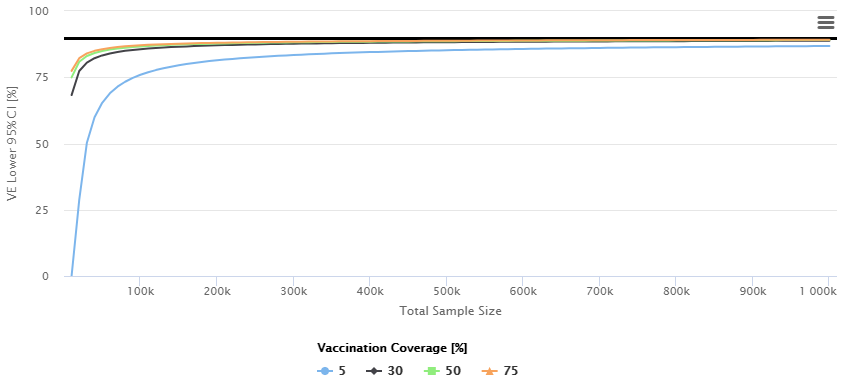


Significant VE should be detectable in subgroups with 10,000+ population (unless vaccine coverage is very low), while subgroup analyses with 100,000+ population should have reasonably high precision. The attack rate will increase over time, raising precision further.

**TNCC**

The plots below show the bottom end of the 95% CI around a VE of 60% and 90% when there are 4 controls per case (i.e. for TNCC 20% are positive and 80% negative) for various coverage figures. (~20% positivity November 2020)

A: 60% VE


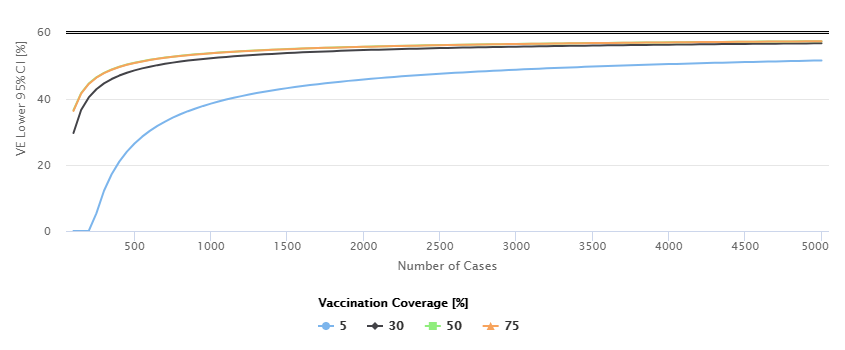


B: 90% VE


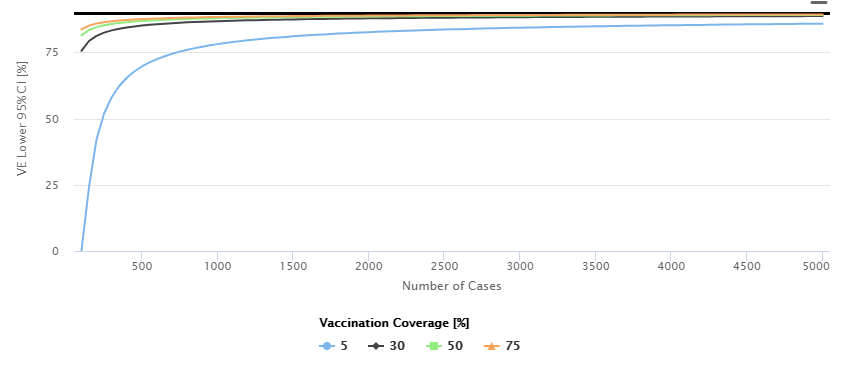


**3.11 Laboratory methods**

**Sentinel specimen collection**

For sentinel swabs individuals (or GPs) take combined throat and nose swabs which are sent by post to the PHE Respiratory Virus Unit at Colindale, North London together with a questionnaire. Pillar testing swabs are taken by individuals or staff at testing site and are tested at multiple labs throughout the country.

**Sentinel testing**

Sentinel samples undergo a molecular analysis for circulating influenza A viruses, influenza B viruses, respiratory syncytial viruses A and B, human metapneumoviruses A and B and other coronaviruses in addition to SARS-CoV-2. Laboratory confirmation is undertaken using RT-PCR assays.

**Other tests used**

A positive SARS-CoV-2 test recorded in GP systems may include results of PCR tests from any laboratory and reported lateral flow test results

**3.12 Statistical analysis**

**Study population characteristics by vaccination status:**

Descriptive statistics will be produced on demographic variables and vaccination status within clinical risk groups.

**Cohort study**

V/E is calculated as 1- RR, where RR is the attack rate of the vaccinated group over the attack rate of the unvaccinated group.

Multivariable Poisson regression will be used with vaccination and week set as time varying covariates and all other variables set according to the individual’s status as of 1^st^ September 2022 (or 2023).

Adjustment for week of swab, age group, sex, risk-status and region (or practice) and will be included at minimum. Inclusion of additional variables including consultation quartile, IMD quintile, ethnicity, smoking status, influenza vaccination status, EFI, large residence, Cambridge Mortality Measure, will be considered individually; variables that do not impact both hospitalisation or death VE by more than 1% need not be included (i.e. the same adjustments will be made for both death and hospitalisation VE).

Vaccination status will be a time-varying categorical variable: 0-13 days post dose, 14-90 days post dose, 91-182 days post dose, with separate categories for vaccine type (and manufacturer if required). Vaccinated 6months+ ago will be the baseline category, to look at the incremental benefit of a new dose.

Person time will be calculated in days and adjusted for as an offset in the model.

The reshape and collapse commands in Stata will be used to aggregate person time and events across the levels of the time varying and fixed covariates.

Cohort analyses will be carried out for both hospitalisation and death outcomes.

Primary cohort analysis will include complete cases only, but unknown categories may be included for some missing data items.

**TND study**

V/E is calculated as 1-OR. Where OR is the odds of vaccination in cases compared to controls (SARS-CoV-2 negative).

Multivariable logistic regression will be used with adjustment for week of swab, age group, risk-status and region (or practice), at minimum. Inclusion of additional variables including sex, consultation quartile, ethnicity, IMD quintile, smoking status, EFI, large residence, Cambridge Morality Measure, and indication for swabbing (grouped as ILI / COVID-like illness, ARI or exacerbation) will be considered individually; variables that do not impact VE by more than 1% need not be included.

Vaccination status will be a categorical variable, captured at the time of swabbing: 0-13 days post dose, 14-91 days post dose, 92-182 days post dose, with separate categories for each vaccine type (or manufacturer if required). Vaccinated 6months+ ago will be the baseline category, to look at the incremental benefit of a new dose.

Primary TND analysis will impute onset status (within/beyond 10 days) where this is not given.

**All studies**

Stratification by age group, risk-group, or variant period will be carried out as required. Interactions between each other factor and vaccination will be tested and if significant stratification will be considered.

Data will be analysed in Stata version 14 or 17.

**4. Limitations**

**4.1 Study population**

Representativeness: the registered practice population is representative of the national population by region, age and gender and the results of the clinical study will be generalisable to the country as a whole.

**4.2 Exposure, vaccination status**

For practical purposes in the UK, the GP record is unlikely to miss vaccination, but since vaccination will be external to the GP, recording delays are likely to occur.

**4.3 Sample size for sub-analysis**

For some stratified or sub-analyses there could be wide confidence intervals.

**4.4 Negative confounding**

These are biases reflecting that high risk groups are more likely to be vaccinated therefore reducing V/E.

This will be addressed by capturing data on risk groups. Nevertheless severity or risk factors and data quality on risk factors could still mean this is not fully measured.

Likewise, if low risk groups – in particular those who have already experienced COVID infection, are less likely to vaccinate, VE could be biased toward negative effectiveness.

This will be addressed by capturing the positive testing history, where possible, and allowing for this in the analysis. We will consider running separate analyses for those with confirmed and/or probable past infection and no history of past infection

**4.5 Positive confounding**

These are biases reflecting a healthy vaccine effect. People with a healthy behaviour and a good functional status are more likely to accept / request vaccination, therefore increasing the measured V/E.

This is not something we will be able to easily capture so is a potential limitation.

**4.6 Other potential limitations**

The comparison of V/E estimates in periods with different COVID-19 activity will allow the study to assess if the observed differences in the occurrence of the selected outcomes in vaccinated and unvaccinated are likely due to the effect of the vaccine or if they reflect baseline differences between the two groups.

Stratification by seasons or wave will be performed to assess this potential bias.

**Antigenic drift / emergence of new strains**

Comparisons of V/E by time period will also inform this.

**Interaction with influenza, causing more severe disease and outcomes**

Dual influenza and COVID-19 infection during the end of the 2019/20 flu season appeared to be associated with increased mortality, hence interaction of influenza and COVID-19 outcomes could be important. The number of co-infections (SARS-CoV-2+, influenza+) appearing in sentinel swab data will be monitored.

**4.7 UKHSA time / workload required**

These large analyses are slow to run. Running of analyses and extraction of results will require approx. 10 full working days.

**5. Dissemination of results**

First V/E estimates will be disseminated among the research team asap.

We would envisage papers to be written for the cohort and TND analysis.

**6. Ethical approval**

The legal basis for conducting this study is Regulation 3 of The Health Service (Control of Patient Information) Regulations 2002, and the work of the Oxford-RCGP RSC with respect to disease surveillance, vaccine effectiveness and safety is specifically approved by UKHSA’s Caldicott Guardian.

**7. Logistical aspects**

The work described here is part of an ongoing programme which will continue according to funding availability.

It is essential that this work continues to ensure data quality and for the entering of swab test results into the records.

It is our expectation that the considerable other costs associated with this project will be absorbed nationally to cover data analysis and report writing.

**Core study teams**

Simon de Lusignan, Rachel Byford, Elizabeth Button, Oxford-RCGP RSC team

Jamie Lopez-Bernal, Heather Whitaker, Nick Andrews, UKHSA.

**8. Key deliverables**

1. Training of practices
   1. Dashboard and observatories to encourage data quality^[[10]](#footnote-10),^^[[11]](#footnote-11)^
   2. Seminar series
   3. Online information at <https://orchid.phc.ox.ac.uk>
   4. Social media
      1. Twitter
      2. Facebook
      3. Linked-in
   5. Work with RCGP and others to raise awareness of the importance of the network, clinical coding etc.
2. Ensuring electronic GP record is updated with swab and vaccine status
   1. Direct feedback to practices by practice liaison team
3. Delivery of data extracts to UKHSA
4. Publicly available data
   1. Vaccine effectiveness papers – led by UKHSA
   2. Ad hoc papers covering areas of mutual interest.

| The subsidiary studies implicit in this proposal will be undertaken in accordance with available funding |
| --- |

**Study 2: Estimation of flu vaccine effectiveness using sentinel practice schemes in the United Kingdom: 2023/24 Proposal**

**1. Background**

Because influenza viruses constantly evolve and vaccines are reformulated every year, VE estimates from previous years cannot be used to estimate VE in the subsequent years. Having annual influenza VE estimates as soon as possible after the start of a seasonal influenza epidemic or pandemic and monitoring it along its course is essential to:

- decide on recommendations for the use of the vaccine
- target complementary or alternative public health measures (e.g. antivirals) for population segments where vaccine is less effective.
- trigger further investigations on seasonal and pandemic vaccines (improve composition, use of adjuvants, need for booster doses).

The UK introduced a universal childhood influenza vaccine programme targeted for 2-16 year old children from 2013-14. In summer 2016, the US Advisory Committee on Immunisation Practice recommended the temporary suspension of use of LAIV in the USA following the CDC assessment of the lack of effectiveness of LAIV in children in the 2015-16 season, with little evidence of significant VE; the suspension was rescinded in spring 2018. Although these results are at odds with those seen in several other countries using LAIV, including the UK, it will be important to ensure the evaluation of the effectiveness of LAIV in children.

In recent seasons, it has become apparent that the current inactivated influenza vaccine programme provides sub-optimal protection to the age-groups >65 years of age, especially against influenza A/H3N2. Adjuvanted vaccines have been preferentially recommended since the 2018/19 season to all in this age-group. Since 2019/20, cell-based quadrivalent vaccines have also been available and used preferentially over standard-dose egg-based vaccines. The recombinant vaccine has also been available since 2021/22, though uptake has been low. In addition, all adults 50-64 years of age have been eligible to receive either cell or egg-based quadrivalent influenza vaccines since the 2020/21 season with the aim of reducing pressure on the health service during the COVID-19 pandemic. It is thus critically important to obtain accurate estimates of the relative effectiveness of these influenza vaccines in the relevant age groups targeted with different vaccine types (2-17, 18-64, 65+), and bringing together data from across the UK will help this aim. This proposal concerns the use of the test-negative case control method to estimate influenza vaccine effectiveness using the various UK sentinel GP swabbing schemes, with a particular focus on the absolute and relative effectiveness of these vaccines.

Further, some influenza positive sentinel swabs are targeted for genetic characterisation. Exploring vaccine effectiveness by sub-clade may help us better understand the impact of antigenic drift on vaccine effectiveness and inform vaccine strain selection. Bringing together this information from sentinel schemes across the UK helps maximise our potential to produce vaccine effectiveness estimates by sub-clade and optimize use of this valuable data.

Finally, a number of RSV vaccines are approaching licensure. There is a need to establish systems to monitor the baseline epidemiology of RSV prior to introduction of these vaccines and for this to then provide a platform to monitor the RSV vaccine programme impact and effectiveness. The burden of disease due to RSV is increasingly being recognised – particularly in young infants and also potentially the elderly, though questions remain. There is a need to establish RSV disease burden in the UK, particularly in younger children and the elderly to inform optimal future use of these new vaccines and to provide a baseline for subsequent impact studies stage.

**2. Aim and Objectives**

**2.1 Aim**

For the 2023/24 season, the aims are to:

- Estimate the pooled influenza vaccine effectiveness for laboratory confirmed infection in primary care across three age groups: children aged 2-17, working-age adults 18-64 and the elderly aged 65+ in the UK.
- Monitor RSV epidemiology in primary care with a focus on children <5 years of age

**2.2 Primary objectives**

The primary objective is to estimate 2023/24 seasonal influenza vaccine effectiveness (VE):

- Overall for any flu type,
- by influenza type (A or B),
- by influenza subtype (A/H1 or A/H3);

within each of 3 broad age groups:

- 2-17 years (and stratified by nasal vs quadrivalent inactivated vaccine),
- 18-64 years of age (and stratified by QIVc vs QIVr vs QIVe),
- >65 years of age (and stratified by aQIV and QIVc).

To describe the epidemiology of RSV in children.

**2.3 Secondary objectives**

To estimate:

- VE by risk status
- VE by influenza main clades/sub-clades
- VE by time since vaccination
- VE by period (depending on circulation patterns)
- VE by prior vaccination status in 2022/23 overall and by type/sub-type (England, Scotland)
- The interaction of age and VE in adults age 18+
- VE against asthma/COPD exacerbation (England only)

**3. Methods**

**3.1 Study designs**

- Test-negative case-control (TNCC) design will be used to estimate VE.
- For RSV descriptive statistics on swab positivity will be collated, by age group, gender, month and risk status.

**3.2 Study population and data source**

**Definitions**

The study will be undertaken in the registered population of sentinel general practice surveillance networks across the UK that undertake respiratory swabbing, details of which have been outlined previously. The surveillance schemes are: Royal College of General Practitioners (RCGP) Research and Surveillance Centre (RSC), Public Health Wales Communicable Disease Surveillance Centre Sentinel GP Surveillance, Public Health Scotland (PHS) CARI surveillance programme.

The study population will be patients presenting to their general practitioner (GP) during the 2023/24 influenza season study period with an acute respiratory illness fitting the case definition (influenza-like illness [ILI] or an acute respiratory infection [ARI]). A registered patient will be fully registered (not temporary) with a valid NHS or CHI number and no qualification period.

ILI is defined as an individual presenting in primary care with an acute respiratory illness with physician-diagnosed fever or complaint of feverishness in the previous 7 days. Acute is defined as sudden onset of symptoms.

Cases will be patients who test positive for influenza A or B virus by real-time PCR.

Controls will be patients with the same symptoms who tested negative for influenza A and B, and negative for SARS-CoV-2.

**Study Site Settings**

| English RCGP/UKHSA scheme:  Patients who contacted their GP, where the GP was part of the Royal College of General Practioners Research and Surveillance Centre (RCGP-RSC) sentinel scheme, and who had a sentinel respiratory swab taken with a result for influenza during the 2023/24 influenza season. Indications for swabbing are influenza-like illness, COVID19-like illness, an acute respiratory infection or exacerbation of asthma/COPD with onset within the last 10 days. Patients are either swabbed and consented by their GP as part of a face-to-face consultation, or directed to take a postal self-swab by GPs through takeatestuk.com after being issued a voucher code. All sentinel swabs are tested for respiratory infections including influenza and RSV using RT-PCR at UKHSA’s Virus Reference Laboratory. Vaccination data are available via linkage to GP records. |
| --- |

| Scottish PHS scheme:  Patients who contacted their GP, where the GP was one of around 140 sentinel practices taking part in the CARI surveillance programme, and who had a respiratory swab taken with a result for influenza during the 2022/2023 influenza season. Indications for swabbing were acute respiratory infection (ARI) with sudden onset within the last 7 days, and aligning with the ECDC definition of ARI. Patients are either consented and swabbed by their GP face-to-face, or they use a self-swabbing kit to take the swab themselves. All sentinel swabs are multiplex tested for 10 respiratory infections at the West of Scotland Specialist Virology Centre and results are held by ECOSS (national database of all virology test results). Vaccination data are available for adults only from the Vaccine Management Tool (VMT). All date can be record-linked using the CHI number. |
| --- |

| Welsh scheme:  Patients who contacted their GP, where the GP was part of the Public Health Wales Sentinel GP Sentinel GP Surveillance Network, and who had a sentinel respiratory swab taken with a result for influenza during the 2022/23 influenza season. Indications for swabbing are influenza-like illness, COVID19-like illness or an acute respiratory infection with onset within the last 10 days. Patients consulting in person are swabbed and consented by their GP as part of a face-to-face consultation or provided with a postal self-swab kit by GPs if consulting via telephone. All sentinel swabs are tested for respiratory infections including influenza and RSV using RT-PCR at Public Health Wales Specialist Virology Centre. Vaccination status is provided by the GP at time of consultation, either sourced from the patients’ medical records or self-reported by the patient to the GP. |
| --- |

**3.3 Study period**

The start of the influenza vaccination campaign is usually in mid-September as soon as influenza vaccine becomes available. It is theoretically available until supplies run out, although the bulk is delivered in late September through to November. The bulk of childhood vaccinations in Primary schools are delivered October to December.

The study period will commence from 4 September 2023 (the point when influenza vaccination rollout and influenza and RSV transmission may begin) and continue for the duration of the UK influenza season. An early calculation of VE will be performed prior to the February 2024 WHO strain selection committee meeting, with a full analysis carried out at the end of the season.

This proposal relates to an ongoing programme of disease surveillance. The end of the influenza season is not fixed *a priori*. The period of influenza activity is determined using data from the national influenza surveillance system.

RSV statistics will be collated at the end of the influenza season using the same season definition as for influenza.

**3.4 VE exposure**

**Definition**

Patients will be defined as vaccinated if they have received the 2022/23 seasonal vaccine at least 14 days before the date of the swab. Note that 2 doses of LAIV are recommended for some children, but will be considered vaccinated from 14 days after the first dose.

**Ascertainment**

Vaccination history is based on electronic health records, which may be supplemented by vaccination information given on the swabbing form if available. Vaccination dates are required, ideally accompanied by vaccine type (LAIV-intranasal; injectable-QIVe, injectable-cell-based QIVc, injectable-egg-based QIVe injectable recombinant QIVr and adjuvanted injectable-aQIV).

**3.5 VE outcome(s)**

The PHS definition of ARI (sudden onset within last 7 days) is –

• Sudden onset of symptoms

• At least one of the following four respiratory symptoms:

o Cough

o Sore throat

o Shortness of breath

o Coryza

AND

• A clinician's judgement that the illness is due to an infection.

The RCGP and Welsh scheme suggest swabbing with onset within the last 10 days, this was extended during the COVID-19 pandemic. However, influenza positivity is highest within the first 7 days, so analyses will aim to restrict to the 0-7 day post onset window.

For the 2023/24 season RCGP are broadening their definition for swabbing to include asthma and COPD exacerbations. These swabs will be excluded from main analyses (where known), and explored in secondary analyses if there are sufficient swab numbers.

Cases are defined as patients with a positive test for influenza, while those with a negative test for influenza and SARS-CoV-2 (but who could have tested positive for other pathogens) are classified as controls.

**3.6 VE exclusion criteria**

Registered patients may be excluded if they have expressed a wish to be excluded from the surveillance programme; or opted out of sharing their data. However, those who consent to a virology swab will include in their consent record sharing for this purpose.

We ideally look for swabs to fall within 0-7 days of reported illness onset. However, patients will not be excluded where no onset date is given or if the number of days between onset and swab exceeds 7 days (the impact of missing and longer onsets will be explored and multiple imputation methods used). Swabs with implausible onset dates post sample receipt will be retained and the onset date replaced as missing.

Patients must have both a negative influenza A and an influenza B result present to be included as a control.

Patients with nonsensical vaccination histories (e.g. outside the flu season, adults that received LAIV, children that received aQIV) or swab dates (e.g. if swabbed after date of receipt) will be excluded, as will swabs received >21days after the reported date taken.

The study will include only those with known age between 2 and 105 and known sex.

Patients residing outside the boundaries of their respective schemes will be excluded i.e. RCGP/UKHSA patients must be resident in England, PHS patients must be resident in Scotland.

Patients with unknown vaccination status will be excluded. This will include patients with no linkage to either NIMS or a GP record in England, and patients for whom no definite yes/no response to the question on vaccination status was recorded on the Welsh swabbing form. Scottish patients will be assumed to be unvaccinated if no record of vaccination is found.

LAIV-eligible patients aged 2-17 will be excluded if they were vaccinated 0-20 days before symptom onset, or if no vaccination date is available, to ensure positives are not related to LAIV. Patients aged 18+ will not be considered fully vaccinated until 14-days post vaccination. Those swabbed during days 1-13 post vaccination will be included in analyses and assigned a separate exposure category for recent vaccination. Vaccinated adults aged 18+ will not be excluded if no vaccination date is available, instead a multiple imputation approach will be used.

**De-duplication**

It is important to ensure that each individual positive episode is included only once, but given that a patient may experience multiple influenza episodes each season, patients may contribute more than one swab to the study, if swabs can reasonably be considered independent.

Patients who had had more than one test can be included as cases and controls more than once, unless the tests were within 28 days. For repeat tests within 28 days, the second test will be excluded, unless a positive test came after a negative test, in which case the negative result will be excluded.

**3.7 Sub-groups**

**Risk groups**

Study subjects are categorised according to Department of Health defined risk categories for influenza vaccination. High risk is determined by the presence of well recognised risk morbidities recorded in the electronic health record for the patient concerned.

- Underlying heart disease
- Chronic respiratory disease
- Diabetes mellitus
- Chronic kidney disease
- Chronic neurological disease
- Splenic dysfunction
- Immunosuppression
- Morbid obesity (BMI>40)
- Chronic liver disease

**Currently pregnant**

- Pregnancy

**Previous vaccination**

Influenza vaccination in the previous season (2022/23)

**Virus genetic characterisation**

Virus clade, and for influenza B lineage

**3.8 Data items**

Most data items are available through electronic health or laboratory records. Some data items are collected by the GPs in their GP practice (or by individuals if self-swabbing) and entered onto a swab questionnaire.

**Table 1: Data items requested**

| **Column** | **Responses** | **Explanation** |
| --- | --- | --- |
|  |  |  |
| Year |  | Year of sample |
|  |  |  |
| Lab_Number |  | Local unique laboratory number |
|  |  |  |
| RECEIPT_DATE | dd/mm/yyyy | Date of sample receipt by lab |
|  |  |  |
| SAMPLE_DATE | dd/mm/yyyy | Date sample taken |
|  |  |  |
| ONSET_DATE | dd/mm/yyyy | Date of onset of illness |
|  |  |  |
| PRESENCE_OF_FEVER | #inp | Not completed on form |
|  | No | No |
|  | Not known | Not known |
|  | Yes | Yes - fever >38 degrees with onset in previous 7 days |
|  |  |  |
| PRESENCE_OF_COUGH | #inp | Not completed on form |
|  | No | No |
|  | Not known | Not known |
|  | Yes | Yes - cough with onset in previous 7 days |
|  |  |  |
| PRESENCE_OF_LOSS_OF_TASTE_OR_SMELL | #inp | Not completed on form |
|  | No | No |
|  | Not known | Not known |
|  | Yes | Yes - cough with onset in previous 7 days |
|  |  |  |
| PRESENCE_OF_SHORTNESS OF BREATH | #inp | Not completed on form |
|  | No | No |
|  | Not known | Not known |
|  | Yes | Yes - SOB with onset in previous 7 days |
|  |  |  |
| PRESENCE_OF_WHEEZE (<5 year) | #inp | Not completed on form |
|  | No | No |
|  | Not known | Not known |
|  | Yes | Yes - wheeze with onset in previous 7 days |
|  |  |  |
| Individual_ID |  | Pseudonomised Individual Identifier |
|  |  |  |
| SEX | m | male |
|  | f | female |
|  | nk | non known |
|  |  |  |
| DATE_OF_BIRTH | dd/mm/yyyy | Date of birth |
|  |  |  |
| CMO_RISKGRP | #inp | Not completed on form |
|  | No | No |
|  | Not known | Not known |
|  | Yes | Yes |
|  |  |  |
| Seasonal_Vaccine_date_23_24 | dd/mm/yyyy | Date of vaccination |
|  |  |  |
| Seasonal_Vaccination_23_24 | #inp | Not completed on form |
|  | Yes | Vaccinated |
|  | nk | Not known |
|  | No | Not vaccinated |
|  |  |  |
| Vaccination_Route_23_24 | #inp | Not completed on form |
|  | Intramusuclar | Vaccinated |
|  | Intranasal | Not known |
|  | nk | Not known |
|  |  |  |
| Vaccine_Type_23_24 | LAIV | LAIV |
|  | aQIV | QIV - adjuvanted |
|  | QIVr | QIV - recombinant |
|  | QIVe | QIV - egg based |
|  | QIVc | QIV - cell based |
|  | nk | Not known |
|  | #inp | Not completed on form |
|  |  |  |
| Past_Seasonal_Vaccination_22_23 | #inp | Not completed on form |
|  | nk | Not known |
|  | Yes | Vaccinated |
|  | No | Not vaccinated |
|  |  |  |
| COVID19_Autumn23_Vaccine_Date | dd/mm/yyyy | Date of any COVID19 vaccination since Sept 2023 |
|  |  |  |
| COVID19_Autumn23_Vaccination | #inp | Not completed on form |
|  | nk | Not known |
|  | Yes | Vaccinated since Sept 2023 |
|  | No | Not vaccinated |
|  |  |  |
| FLU_PCR_Result | #flub | Influenza B |
|  | #h1 | Influenza AH1 |
|  | #h3 | Influenza AH3 |
|  | #flua | Influenza A(unknown) |
|  | #h1h3 | Influenza AH1 AND influenza AH3 |
|  | #h1h3flub | Influenza AH1 AND influenza AH3 AND influenza B |
|  | #h3flub | Influenza AH3 AND influenza B |
|  | #h1flub | Influenza AH1 AND influenza B |
|  | #laiv | LAIV strains detected |
|  | #nvd | No virus detected |
|  |  |  |
| Other_PCR_Result | #covid | SARS-CoV-2 |
|  | #hmpv | Human Metapneumavirus |
|  | #ra | RSV A |
|  | #rb | RSV B |
|  | #rarb | Both RSV A and RSV B detected |
|  | #rsv | RSV untyped |
|  | #adeno | Adenovirus |
|  | #rhino | Rhinovirus |
|  | #corona | Seasonal coronavirus |
|  | #nvd | No virus detected |
|  |  |  |
| Final_Overall_Result | #b,#nvd | Influenza B |
|  | #h3,#nvd | Influenza AH3 |
|  | #h1,#nvd | Influenza AH1 |
|  | #nvd,#covid | SARS-CoV-2 |
|  | #nvd,#hmpv | Human Metapneumavirus |
|  | #nvd,#ra | RSV A |
|  | #nvd,#rb | RSV B |
|  | #nvd,#rarb | Both RSV A and RSV B detected |
|  | #nvd | No virus detected |
|  | Results awaited | Results awaited |

Some of the above data items above are not essential to the main study, such as symptom information, these may be used if there is specific interest in symptom groups or to exclude those with COVID19-like illness. The level of detail could be lessened to enable data sharing, e.g. age on 1^st^ September 2023 can be given instead of DOB, indication that vaccination was given ≥0, 14 and 21 days prior rather than precise vaccination dates, lab number can be pseudonymised.

Further genetic characterisation data is requested, where available, for the 2022/23 season circulating strains of interest include:

| genetic group | Like virus | amino acid substitutions |
| --- | --- | --- |
| H1N1 |  |  |
| 6B.1A.5a.2 | A/Victoria/2570/2019 (vaccine) | K130N, N156K, L161I, V250A |
| 6B.1A.5a.2a | A/Sydney/5/2021 | K54Q, A186T, Q189E, E224A, R259K, K308R |
| 6B.1A.5a.2a.1 | A/Norway/25089/2022 | P137S, K142R, D260E |
| 6B.1A.5a.1 | A/Guangdong-Maonan/SWL1536/2019 |  |
| H3N2 |  |  |
| 3C.2a1b.2a.2 | A/Bangladesh/4005/2020 | Y159N, T160I, L164Q, G186D, D190N, Y195F |
| 3C.2a1b.2a.2a | A/Darwin/9/2021 (vaccine) | H156S |
| 3C.2a1b.2a.2a.1 | A/Slovenia/8720/2022 | D53G, D104G, K276R |
| 3C.2a1b.2a.2a.1a |  | L157I, K220R |
| 3C.2a1b.2a.2a.1b | A/Catalonia/NSVH161512067/2022 | I140K, R299K |
| 3C.2a1b.2a.2a.2 | A/Poland/97/2022 | D53G, R201L, S219Y |
| 3C.2a1b.2a.2a.3 | A/Norway/24873/2021 | D53N, N96S, I192F |
| 3C.2a1b.2a.2a.3a |  | E50K |
| 3C.2a1b.2a.2a.3a.1 |  | I140K |
| 3C.2a1b.2a.2a.3b |  | I140M |
| 3C.2a1b.2a.2b | A/Thuringen/10/2022 | E50K, F79V, I140K |
| 3C.2a1b.2a.2c |  | S205F, A212T |
| 3C.2a1b.2a.2d |  | G62R, H156Q, S199P |
| 3C.2a1b.2a.1a | A/Cambodia/e0826360/2020 |  |
| B |  |  |
| V1A.3a.2 | B/Austria/1359417/2021 (vaccine) | A127T, P144L, K302R |
| V1A.3a.2 (i) |  | T182A, D197E, T221A |
| V1A.3a.2 (ii) |  | E128K, A154E, S208P |
| V1A.3a.2 (iii) |  | E198G |
| V1A.3a.2 (iv) |  | D129G, D197E |
| V1A.3a.2 (v) |  | R80G, E184K |
| V1A.3a.2 (vi) |  | E183K |

**3.9 Laboratory methods**

**Specimen collection**

GPs take combined throat and nose swabs which will be sent from sentinel GP surveillance networks to the usual laboratory.

**Tests used**

Influenza laboratory confirmation will be undertaken using comparable real-time PCR assays. Samples undergo a molecular analysis for circulating influenza A and influenza B viruses, respiratory syncytial viruses A and B, human metapneumoviruses A and B and COVID-19. Further analyses including that for seasonal coronaviruses, rhinovirus, adenovirus may be carried out. Further influenza genetic characterisation work is undertaken to determine genetic clade, and for influenza B viruses the lineage.

**3.10 Statistical Analysis**

Stata 14/17 (StataCorp, College Station, TX, USA) will be used for this data analysis.

**Flu and RSV descriptive analyses**

Cases and non-cases will be described according to:

- Month of swab – count (%)
- Sex – count (%)
- Age group – count (%)
- Region - count (%)
- Risk status – count (%)
- Vaccination Status including Vaccination Type – count (%)
- Past season vaccination status – count (%)
- COVID-19 vaccination status – count (%)

Differences in swab positivity within these groups will be tested using Chi-squared or Fisher’s exact test, as appropriate. Swabs with a gap from onset to swab >7d, and swabs taken less than 14days (21days for children) of vaccination will not be included in descriptive analyses.

**Flu TNCC**

For analysis of this test-negative case-control study, logistic regression will be used to calculate the unadjusted odds ratios for influenza vaccination in cases compared to controls, with a 95% confidence interval. This will be used to calculate an unadjusted VE as unadjusted VE=1-OR.

Logistic regression will be used to calculate the odds ratio for vaccination, adjusted for week of swab, scheme and age group, and additionally for relevant characteristics which change the vaccine effect by ≥1% (including risk status and sex). This will be used to calculate the adjusted VE. This will be undertaken for all cases/controls in the elderly, working-age adults and children separately, and in relevant subgroup analyses.

**Missing data**

We will aim to use multiple imputation methods for our main analysis where possible, rather than exclude data. However, data on vaccination status, age group and date of swab must be complete for inclusion in the study.

*Multiple imputation for missing risk status*

Multiple imputation methods will be used for missing data on risk status, taking into account patient age, and vaccination history and age eligibility.

*Multiple imputation for missing onset date*

Multiple imputation will be used where onset dates are missing. An interaction term between vaccination status and indicator for swab within 0-7days of onset will fitted (the sample size needs to remain consistent). Where onset is missing this indicator will be imputed based on the proportion of samples with known onset falling within the 7day window, taking into account positivity for flu or other viruses and patient age.

*Multiple imputation for missing vaccination date*

Where an adult patient has indicated that they were vaccinated at the time of the swab, but no vaccination date was given, a hot-deck imputation approach will be used. Vaccination dates will be sampled from other vaccinated patients within the same broad age cohort (18-64 or 65+) and swabbed within the same week. The imputed vaccination dates will be used to assign patients as within 1-13 days of vaccination or fully vaccinated.

In secondary analyses by vaccine type, a category for unknown vaccine type will be included. (Since vaccine type is almost universally missing for patients whose vaccination date is missing, the above imputation approach for vaccination dates need not be taken for analyses of vaccine type).

**Sensitivity analyses**

1. Exclusion of individuals with unknown onset date and missing data items.
2. Restriction to patients with Asthma/COPD exacerbation in RCGP data only, and/or exploration of indicators for swabbing (ARI/ILI/COVID-like illness).
3. Inclusion of SARS-CoV-2 positive cases, with/without adjustment for Autumn 2023 COVID-19 vaccination status.

**Sample size**

All eligible patients will be included. Expected cell counts of vaccinated cases, vaccinated controls, unvaccinated cases, unvaccinated controls will be calculated. If any expected cell count is below 10, the sample size is deemed too small to produce reliable estimates and VE will not be published.

**4. Dissemination of results**

First V/E estimates (intra-seasonal) using the test-negative design will be disseminated among the research team and to WHO as part of the GIVE report early during the influenza season (early Jan of the influenza season) followed by the end of season analysis. We would envisage papers to be written at the end of the season following presentation of results at JCVI and VEBIS.

**5. Ethical approval**

The collection of the clinical data accords with routine practice. Specific ethical approval is not necessary. The analysis of swab forms according to positivity is currently undertaken as part of the routine assessment of the virological swabbing programme. The swabs are taken to assist clinical management.

1. de Lusignan S, Correa A, Smith GE, Yonova I, Pebody R, Ferreira F, Elliot AJ, Fleming D. RCGP Research and Surveillance Centre: 50 years' surveillance of influenza, infections, and respiratory conditions. Br J Gen Pract. 2017 Oct;67(663):440-441. doi: 10.3399/bjgp17X692645.. [↑](#footnote-ref-1)
2. de Lusignan S, Lopez Bernal J, Zambon M, Akinyemi O, Amirthalingam G, Andrews N, Borrow R, Byford R, Charlett A, Dabrera G, Ellis J, Elliot AJ, Feher M, Ferreira F, Krajenbrink E, Leach J, Linley E, Liyanage H, Okusi C, Ramsay M, Smith G, Sherlock J, Thomas N, Tripathy M, Williams J, Howsam G, Joy M, Hobbs R. Emergence of a Novel Coronavirus (COVID-19): Protocol for Extending Surveillance Used by the Royal College of General Practitioners Research and Surveillance Centre and Public Health England. JMIR Public Health Surveill. 2020 Apr 2;6(2):e18606. doi: 10.2196/18606.. [↑](#footnote-ref-2)
3. [↑](#footnote-ref-3)
4. de Lusignan S. Codes, classifications, terminologies and nomenclatures: definition, development and application in practice. Inform Prim Care. 2005;13(1):65-70. doi: 10.14236/jhi.v13i1.580. [↑](#footnote-ref-4)
5. de Lusignan S, Liyanage H, McGagh D, Jani BD, Bauwens J, Byford R, Evans D, Fahey T, Greenhalgh T, Jones N, Mair FS, Okusi C, Parimalanathan V, Pell JP, Sherlock J, Tamburis O, Tripathy M, Ferreira F, Williams J, Hobbs FDR. COVID-19 Surveillance in a Primary Care Sentinel Network: In-Pandemic Development of an Application Ontology. JMIR Public Health Surveill. 2020 Nov 17;6(4):e21434. doi: 10.2196/21434. [↑](#footnote-ref-5)
6. Cross reference: Footnote No 1: <https://www.gov.uk/government/publications/priority-groups-for-coronavirus-covid-19-vaccination-advice-from-the-jcvi-2-december-2020/priority-groups-for-coronavirus-covid-19-vaccination-advice-from-the-jcvi-2-december-2020>

   Clift AK, Coupland CAC, Keogh RH, Diaz-Ordaz K, Williamson E, Harrison EM, Hayward A, Hemingway H, Horby P, Mehta N, Benger J, Khunti K, Spiegelhalter D, Sheikh A, Valabhji J, Lyons RA, Robson J, Semple MG, Kee F, Johnson P, Jebb S, Williams T, Hippisley-Cox J. Living risk prediction algorithm (QCOVID) for risk of hospital admission and mortality from coronavirus 19 in adults: national derivation and validation cohort study. BMJ. 2020 Oct 20;371:m3731. doi: 10.1136/bmj.m3731. [↑](#footnote-ref-6)
7. de Lusignan S, Dorward J, Correa A, Jones N, Akinyemi O, Amirthalingam G, Andrews N, Byford R, Dabrera G, Elliot A, Ellis J, Ferreira F, Lopez Bernal J, Okusi C, Ramsay M, Sherlock J, Smith G, Williams J, Howsam G, Zambon M, Joy M, Hobbs FDR. Risk factors for SARS-CoV-2 among patients in the Oxford Royal College of General Practitioners Research and Surveillance Centre primary care network: a cross-sectional study. Lancet Infect Dis. 2020 Sep;20(9):1034-1042. doi: 10.1016/S1473-3099(20)30371-6. [↑](#footnote-ref-7)
8. Joy M, Hobbs FR, Bernal JL, Sherlock J, Amirthalingam G, McGagh D, Akinyemi O, Byford R, Dabrera G, Dorward J, Ellis J, Ferreira F, Jones N, Oke J, Okusi C, Nicholson BD, Ramsay M, Sheppard JP, Sinnathamby M, Zambon M, Howsam G, Williams J, de Lusignan S. Excess mortality in the first COVID pandemic peak: cross-sectional analyses of the impact of age, sex, ethnicity, household size, and long-term conditions in people of known SARS-CoV-2 status in England. Br J Gen Pract. 2020 Nov 26;70(701):e890-e898. doi: 10.3399/bjgp20X713393. [↑](#footnote-ref-8)
9. de Lusignan S, Joy M, Oke J, McGagh D, Nicholson B, Sheppard J, Akinyemi O, Amirthalingam G, Brown K, Byford R, Dabrera G, Krajenbrink E, Liyanage H, LopezBernal J, Okusi C, Ramsay M, Sherlock J, Sinnathamby M, Tsang RSM, Tzortziou Brown V, Williams J, Zambon M, Ferreira F, Howsam G, Hobbs FDR. Disparities in the excess risk of mortality in the first wave of COVID-19: Cross sectional study of the English sentinel network. J Infect. 2020 Nov;81(5):785-792. doi: 10.1016/j.jinf.2020.08.037. [↑](#footnote-ref-9)
10. Pathirannehelage S, Kumarapeli P, Byford R, Yonova I, Ferreira F, de Lusignan S. Uptake of a Dashboard Designed to Give Realtime Feedback to a Sentinel Network About Key Data Required for Influenza Vaccine Effectiveness Studies. Stud Health Technol Inform. 2018;247:161-165.. [↑](#footnote-ref-10)
11. Liyanage H, Akinyemi O, Pathirannahelage S, Joy M, de Lusignan S. Near Real Time Feedback of Seasonal Influenza Vaccination and Virological Sampling: Dashboard Utilisation in a Primary Care Sentinel Network. Stud Health Technol Inform. 2020 Jun 16;270:1339-1340. doi: 10.3233/SHTI200431. [↑](#footnote-ref-11)
