# Supplementary material for: Postpandemic Sentinel Surveillance of Respiratory Diseases in the Context of the World Health Organization Mosaic Framework: Protocol for a Development and Evaluation Study Involving the English Primary Care Network 2023-2024
Source: JMIR Public Health Surveill. 2024 Apr 3;10:e52047. doi: 10.2196/52047 (PMC11024753; doi:10.2196/52047)
Supplement: Multimedia Appendix 4 [file publichealth_v10i1e52047_app4.docx]

# Multimedia Appendix 4. Distribution of virology and serology sampling practices and the entire network.


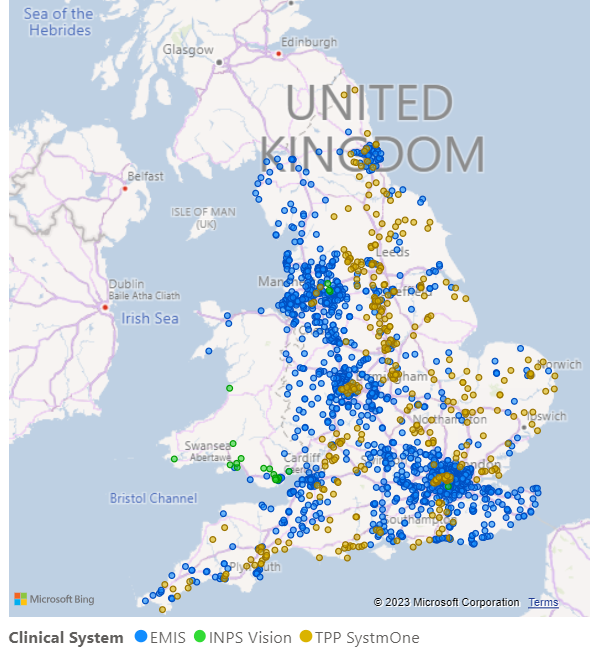


**Figure S1: Distribution of Oxford-RCGP RSC network practices by clinical system**


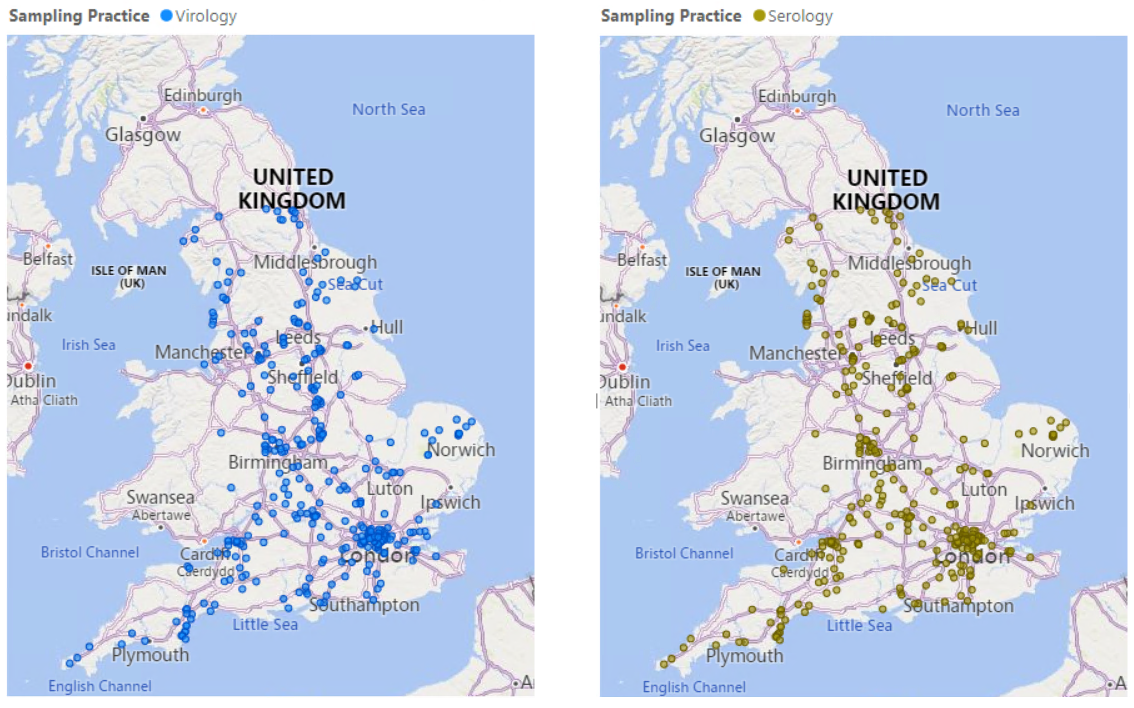


**Figure S2: Distribution of virology and serology sampling practices within the Oxford-RCGP RSC network**
