# Supplementary material for: Postpandemic Sentinel Surveillance of Respiratory Diseases in the Context of the World Health Organization Mosaic Framework: Protocol for a Development and Evaluation Study Involving the English Primary Care Network 2023-2024
Source: JMIR Public Health Surveill. 2024 Apr 3;10:e52047. doi: 10.2196/52047 (PMC11024753; doi:10.2196/52047)
Supplement: Multimedia Appendix 5 [file publichealth_v10i1e52047_app5.docx]

# Multimedia Appendix 5. Practice liaison team communications.

The Practice Liaison Officer (PLO) team are responsible for preparing and delivering communications (“comms”) on behalf of the Research and Surveillance Centre (RSC) and Royal College of General Practitioners (RCGP, RCGP-RSC). Our communications rely on both digital and face-to-face delivery and mediums. The sections below detail the distinct types of communications organised by the PLO team, their frequency, aims, and target audience. We look to communicate key developments and important updates within the RCGP-RSC network.

**1. (Digital) Newsletters**

The weekly and monthly newsletters sit with the PLO team, and are designed to inform, update, and support practices in the surveillance network. The sections below cover our three digital newsletters: (Figure S1.1) **Sampling is Informing**, sent out weekly; (Figure S1.2) **The Director’s Message**, sent out weekly; and (Figure S1.3) **The Monthly Newsletter**, sent out monthly. All email communications are shared with RSC members and RSC-adjacent contacts who have “opted into” our newsletters when joining the network, or at other relevant or appropriate points.

The newsletters are designed to be visually appealing, with clear infographics and concise text. We wish to communicate to our network in a style and format that avoids being burdensome or unnecessarily technical.

**1.1 Sampling is Informing**

The weekly “Sampling is Informing” newsletter covers data from the Weekly Returns Service. The PLO Team sends the newsletter on behalf of its General Practitioner colleagues, namely, Dr Gavin Jamie, Dr Debasish Kar, Dr Rashmi Wimalaratna, Dr Nick Thomas, Dr Will Elson, and Prof Simon de Lusignan. The newsletter covers several areas relevant to sampling, but primarily focuses on the national incidence of infection. We then communicate this information via test, graphs, and infographics.

The newsletter aims to support our principle of “sampling is informing” – the more virology and serology samples we can collect, the better representation, accuracy and information we can offer of viral spread in England. The newsletter also serves to encourage virology and serology sampling among our network by sharing numbers and swabbing rates across the country. These figures are also drawn up by the PLO team. Below is an excerpt from our Sampling is Informing newsletter.


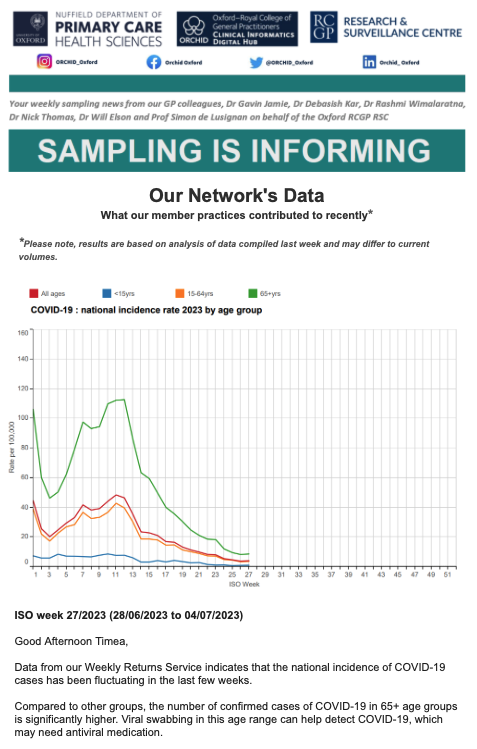

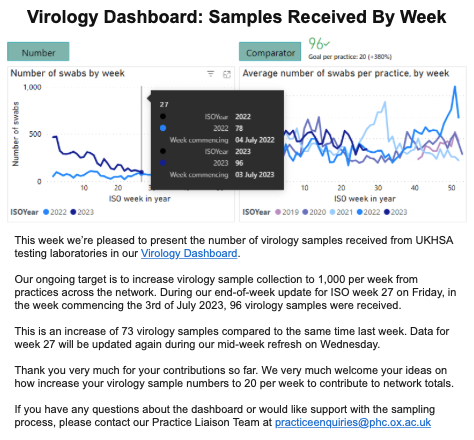


Figure S1.1: Sampling is Informing Newsletter, Excerpt

**1.2 Director’s Message**

Our Director’s Message is a weekly newsletter, designed to communicate updates from and on behalf of Simon de Lusignan, the director of the RCGP-RSC. It is intended to offer a personable communication to practices within the network and send updates, changes, and round of the week with a Friday afternoon message. As of July 2023, it is sent out to over 2,000 RSC-RCGP contacts. Below are examples and excerpt from our Summer 2023 Director’s Message.


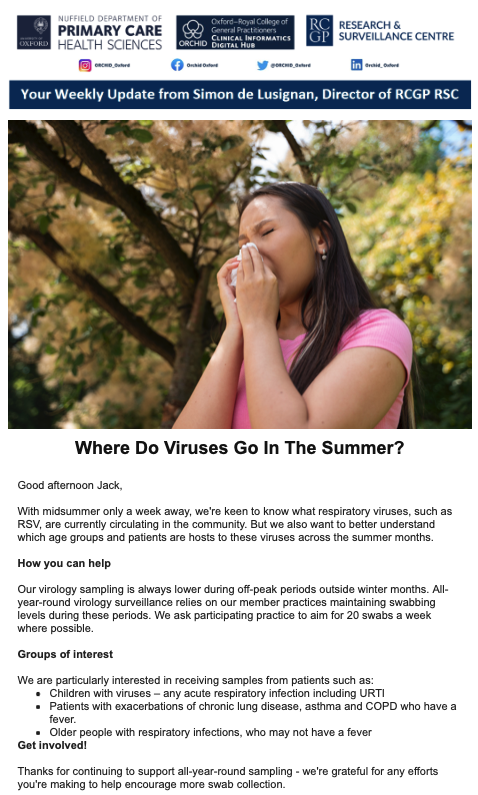

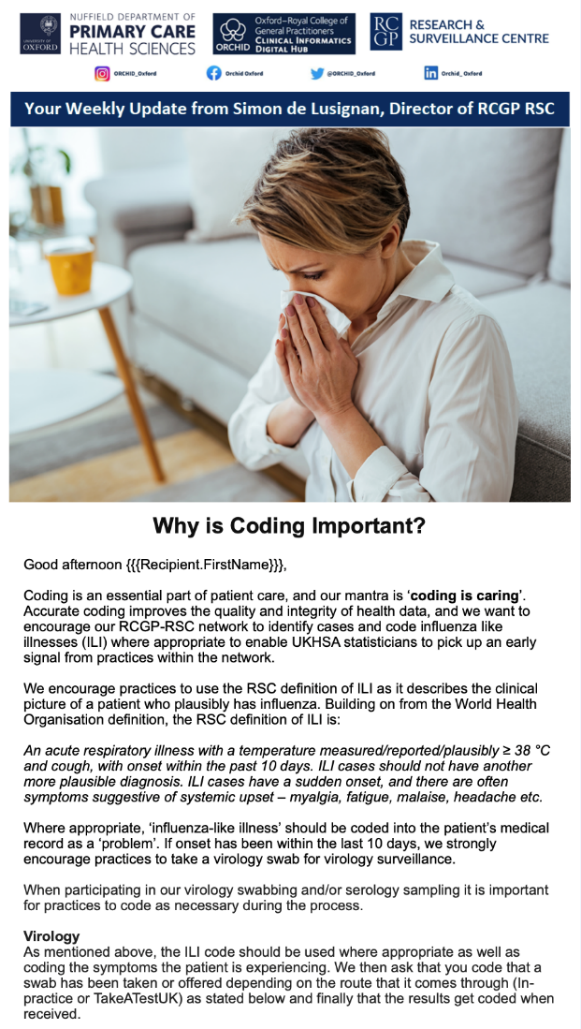

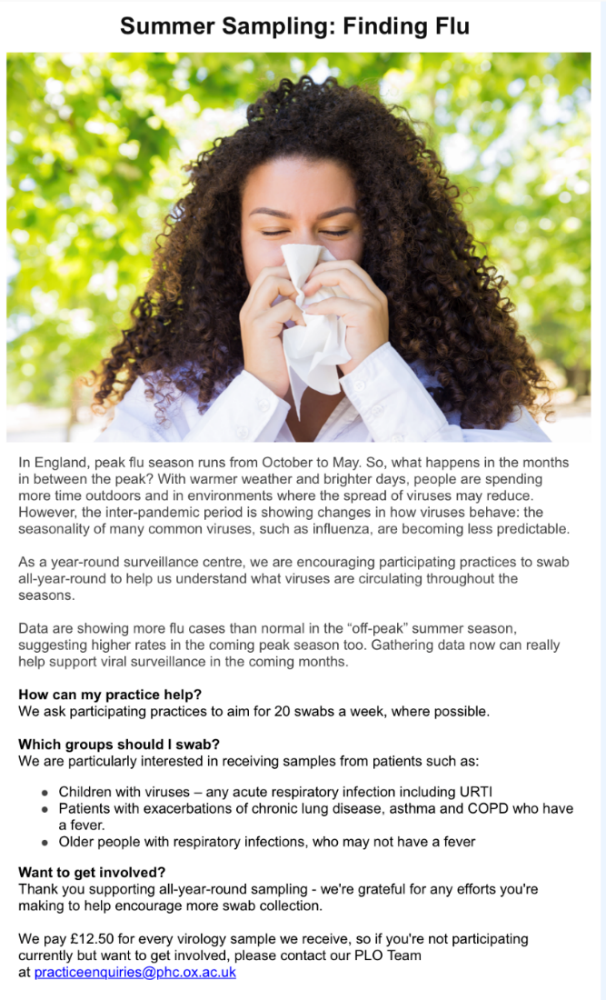


Figure S1.2: Director’s Message Newsletter, Excerpt

**1.3 Monthly Newsletter**

The Monthly Newsletter presents a retrospective of and reflection on the previous month. Here the focus is primarily on presenting and highlighting our main news, practice-facing communications, and ongoing studies in our network. Overall, we find that maintaining regular contact with practices helps support them in the surveillance work they contribute.


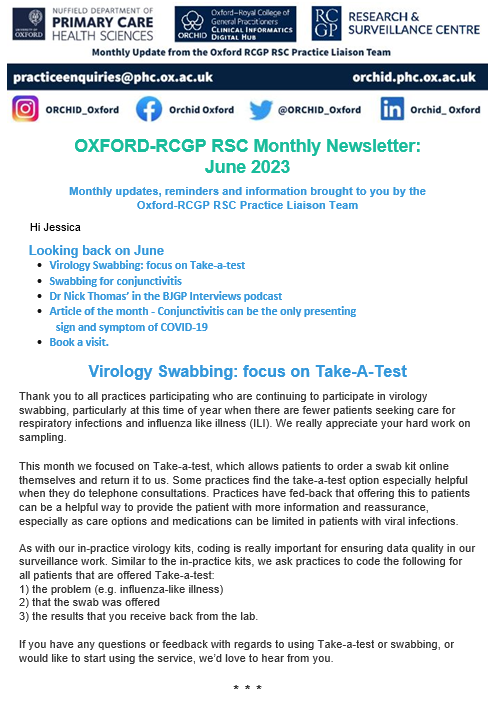


Figure S1.3: Monthly Newsletter, Excerpt

**2. Online Courses and Webinars**

Our FORT Series run on the RCGP/RSC YouTube webinar series. Engaging and informative, they aim to provide a high-level overview of topics relevant to engaging in surveillance. Examples include ‘The Importance of Virology in a Year of Respiratory Illness Reset’ and ‘The Value of Serology Sampling.’


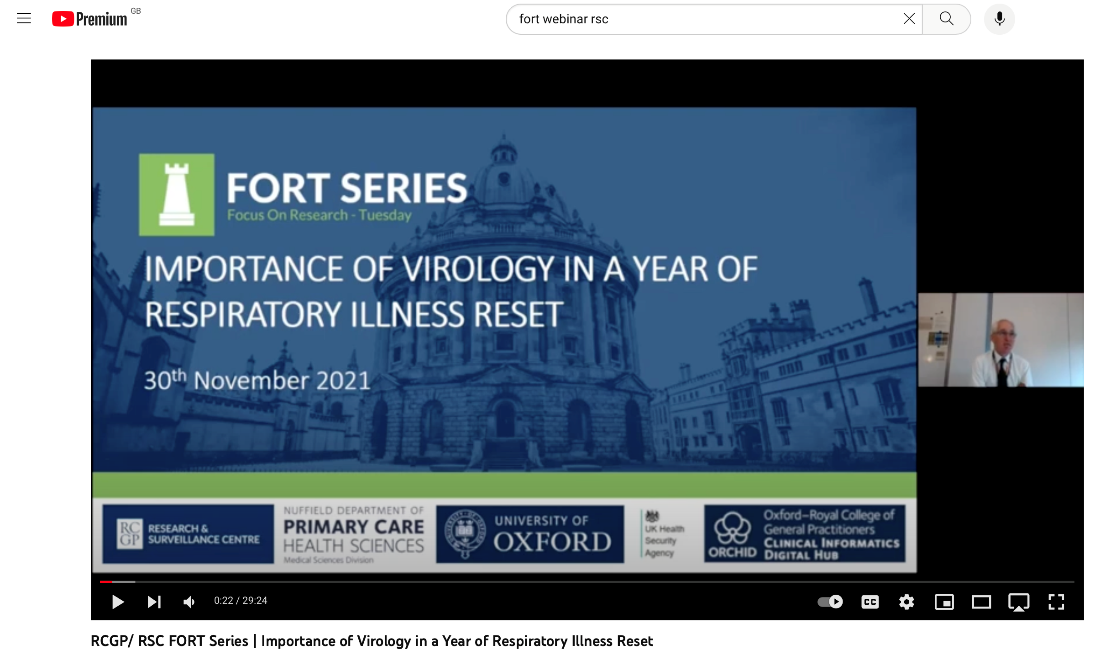


Figure S2.1: Fort Series Webinar

**3. ORCHID Website** (<https://orchid.phc.ox.ac.uk/surveillance/surveillance)>

Our website contains important information and links for practicing both within our network and those looking to join. Especially popular are our surveillance dashboards: practices within the network can enter in their “key” and track their swabbing by different categories. These apply to both serology and virology and are updated regularly with data inputted by the RCGP-RSC's data team.

Key links:

[**How to Join**](https://orchid.phc.ox.ac.uk/surveillance/how-to-join)

[**Material Request**](https://orchid.phc.ox.ac.uk/surveillance/material-request)

[**Study – Expression of Interest**](https://orchid.phc.ox.ac.uk/surveillance/study-2013-expression-of-interest)

[**UKHSA Surveillance Commissioning Letter**](https://orchid.phc.ox.ac.uk/files/resources/rcgp_ukhsa_2022_23_surveillance_letter_final_sept2022_v2.pdf)

[**Swabbing for Conjunctivitis Guide**](https://orchid.phc.ox.ac.uk/files/resources/rcgp-rsc-guide-swabbing-for-conjunctivitis.pdf)

Resources for Patients:

[**Serology Sampling Patient Information Sheet**](https://orchid.phc.ox.ac.uk/files/resources/serology-sampling-patient-information-sheet.pdf)

[**Virology Swabbing Patient Information Sheet**](https://orchid.phc.ox.ac.uk/files/resources/virology-swabbing-patient-information-sheet.pdf)

**4. Face to Face (F2F) Outreach**

Finally, the PLO team is trained to offer outreach to practices through a variety of face-to-face mediums. The aim is to engagement practices and the wider primary care infrastructure.

These are, but not limited to:

- Practice calls and visits
- Campaigns to encourage practice visits
- Attending forums and conferences to give talks, presentations, or attend to stalls
- Live demonstrations of virology and serology packs.

These are designed to either introduce a new practice into the RSC network or advise on further research. Building relationships with our member practices. Encouraging swabbing and sampling through visits, answer questions, empower network, support concerns. Practice visit form.
