# Supplementary material for: Postpandemic Sentinel Surveillance of Respiratory Diseases in the Context of the World Health Organization Mosaic Framework: Protocol for a Development and Evaluation Study Involving the English Primary Care Network 2023-2024
Source: JMIR Public Health Surveill. 2024 Apr 3;10:e52047. doi: 10.2196/52047 (PMC11024753; doi:10.2196/52047)
Supplement: Multimedia Appendix 6 [file publichealth_v10i1e52047_app6.docx]

# Multimedia Appendix 6. Summary of a pilot study to introduce pediatric phlebotomy training in the Research and Surveillance Centre network by region.

**Aim**

To improve serology sampling rates in under 15s, with a focus on under 8s in the RSC-RCGP network. We look to improve this via training in primary health care settings.

Results from this study will help contribute towards testing for vaccine efficacy across the English population.

**Background and Rationale**

Figures on serology sampling by age band consistently show under 15s and under 8s underrepresented across the network. In turn, the data do not provide a fully representative picture of vaccine efficacy in the English population across age groups.

Fortunately, our network contains practices who run successful and efficient paediatric blood sampling clinics and training. We will look to these practices for guidance on how best to introduce and improve phlebotomy in practices across the network (see p.3 for a summary of findings from practice interviews).

The feedback from these practices will inform our rationale and approach moving forward. Specifically, we are looking for (paediatric) phlebotomy guidance that is transferable across the RSC network.

**Practice Recruitment for Paediatric Phlebotomy**

The pilot aims to recruit fourteen England-based practices in total, with two practices recruited from each NHS region:

• North-East and Yorkshire region.

• North-West region.

• Midlands region.

• East of England region.

• South-West region.

• South-East region.

• London region.

**Collecting Blood Samples**

From September, and in time for flu season, we will move to incorporate our new blood sampling “cap,” distributed by age group/band (see Table 1). The network receives a good volume of blood samples but would benefit from an increase in the under 16s band.

**Table 0-17: Monthly Blood Sample Quota for entire network, by age group and sample number**

| **Age group** | **Number of blood samples per month** |
| --- | --- |
| Children (aged 15 years and younger) | 500 samples |
| Adults (“working age”) | 500 samples |
| Adults (>/=65) | 500 samples |

Practices will be asked to focus on sampling children. Informed consent will be taken verbally from the parents to obtain a second, voluntary serological samples from children when they attend for a prearranged blood test. Practices will be asked to ensure that parents will be present at the time of collecting samples. For other age groups, verbal consent will be taken, as is done for usual serological sampling.

**Pricing per age band**

Practices will return blood samples and a lab form, indicating the age of the patient. The RSC will reimburse practices for the additional time spent on blood sampling. By age band, we will offer the following payments:

**Table 18-64: Payments for Blood Samples, by Age Group**

| **Age group** | **Payment per sample** |
| --- | --- |
| Children (aged 8 years and younger) | £30 |
| Adolescents | £11 |
| Adults (>/=18) | £5.50 |

We may introduce more segmentation of age groups for the pilot.

**Training for Improved Phlebotomy Samples**

Following conversations with practices, we have identified training as essential for increasing paediatric phlebotomy. With the help of existing literature and discussions with practices already doing paediatric bloodwork within the network, we will put together a pilot.

**Summary**

We are looking to run a pilot of fourteen practices across seven English regions, with two general practices recruited per region. We intend to begin a sampling cap in September which will reduce “oversampling” in particular age groups. Payment per sampling should serve as an incentive to increase sampling in children and adolescents. By providing training to practices, it will help support and streamline their operations. The Practice Liaison Team will be responsible for coordinating the pilot.

Paediatric Phlebotomy: Pilot Study Discussions

“Drawing blood from children requires a gentle touch and compassionate approach”

– The Phlebotomy Centre

Summary of Meeting with Woodbridge Practice

**Costing:** At £30 payment per blood sample, the practice [“they”] can imagine the higher payment accommodating and covering the cost of longer appointments, additional work, and running extra clinics.

**Training:** If included in the pilot, they would appreciate the training. They would not be able to accommodate the pilot without the RSC supporting training in paediatric phlebotomy.

**Time:** The staff describe a need for longer appointments and time to prepare for the appointments beforehand too.

Regarding longer appointments, the practice suggests a time of day that would work best for them too. The practice recommends offering an end-of-session slot for children to do bloodwork. For instance, this would be after school “when the children are watered and fed,” and there are fewer people and patients in the practice, allowing more time to ensure children are comfortable, and there is enough space to accommodate attending parents and children.

Regarding preparing for appointments, the staff discuss the importance of creating a supportive environment for bloodwork. The nurse manager and practice nurse explain that ten-minute appointment slots would not offer enough time to build rapport and “gain parent and child trust.”

**Materials needed:** The phlebotomist suggests that smaller bottles would be needed for this pilot and agrees that butterfly needles would be helpful too.

**Sample size:** The practice describes 5-6 additional weekly samples as a realistic addition for paediatric phlebotomy.

**Communications:** A telephone call system whereby the practice gives parents “a heads up about the pilot” and to give “that little bit of reassurance.” The practice describes relying on their texting system as an effective way of contacting parents to ask for extra sample.

**Patient consent:** Always talking with parents and gaining their consent, verbal consent only.

**Training for pilot:** having started a new study so at capacity. However, they do have two healthcare assistants who are interested in paediatric phlebotomy training.

**Notes, comments, reflections:**

Woodstock Bower Surgery presents an example of a practice that seeks to use its money from the (extremely high) volume of samples they were taking to hire a full-time phlebotomy nurse who could up the volume of paediatric sampling amongst other things. They also talked about using the butterfly needle to draw from younger patients. The PLO team believes that they present a good case of a practice that could provide qualitative data/feedback on blood work in children.
