# Supplementary material for: Postpandemic Sentinel Surveillance of Respiratory Diseases in the Context of the World Health Organization Mosaic Framework: Protocol for a Development and Evaluation Study Involving the English Primary Care Network 2023-2024
Source: JMIR Public Health Surveill. 2024 Apr 3;10:e52047. doi: 10.2196/52047 (PMC11024753; doi:10.2196/52047)

# Multimedia Appendix 7. An introduction to LabLinks.

Lablinks is the name that has been given to a new workflow that the University is looking to introduce to improve the way in which surveillance samples are requested and subsequently received by the performing laboratory.

**Current approach:**

Currently when a participating GP Practice engages with a patient who may be suitable for one of the surveillance programs, a paper form is completed containing the patient detail and additional necessary information. A sampling kit that has been supplied directly to the practice is then used to collect the sample. The completed kit and form are then posted directly to the performing laboratory by the practice.


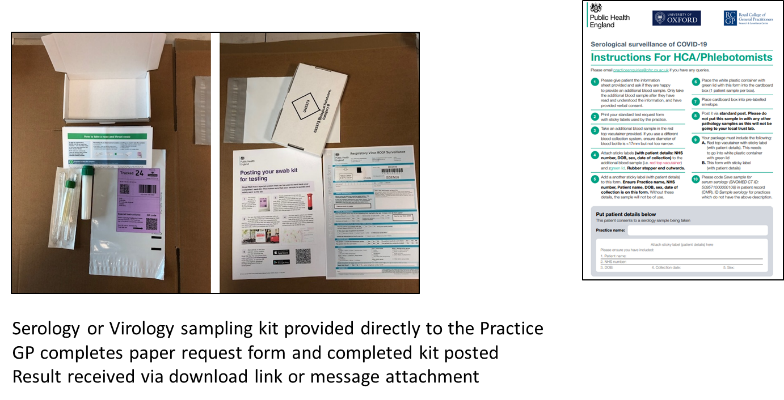


Shortcomings of this process are that it does not align with the standard mechanism a GP would use to collect a sample for diagnostic purposes. It is time consuming for the GP to complete the form and the path the sample follows is different to those samples taken for routine work. It is felt that this variation in process is one of the reasons the uptake of sampling amongst participating practices has not been greater.

**LabLinks approach:**

LabLinks will utilise the existing relationship that the GP Practice has with its local performing Laboratory. This relationship will include connectivity between the GP Practice system (Emis Web, TPP SystmOne) and an Order Comms solution (ICE Desktop, tQuest, Dart or CyberLab). It will include an existing mechanism for the supply of sample containers as well as an established courier process to facilitate the collection of the samples and transport to the Laboratory.


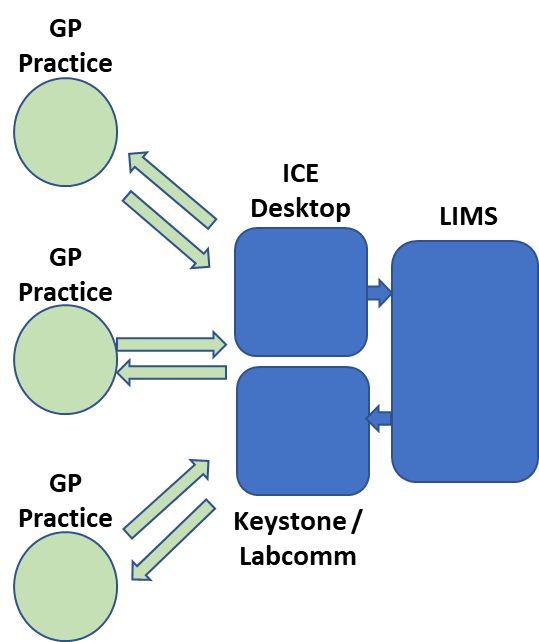


ICE Desktop: [www.clinisys.co./uk/en/healthcare/clinisys-order-results-management/](http://www.clinisys.co./uk/en/healthcare/clinisys-order-results-management/)

X-Lab: <https://x-labsystems.com/products/labgnostic>

LabLinks will engage with the local Laboratory and agree the creation of a “RCGP Surveillance” panel within the Order Comms solution.


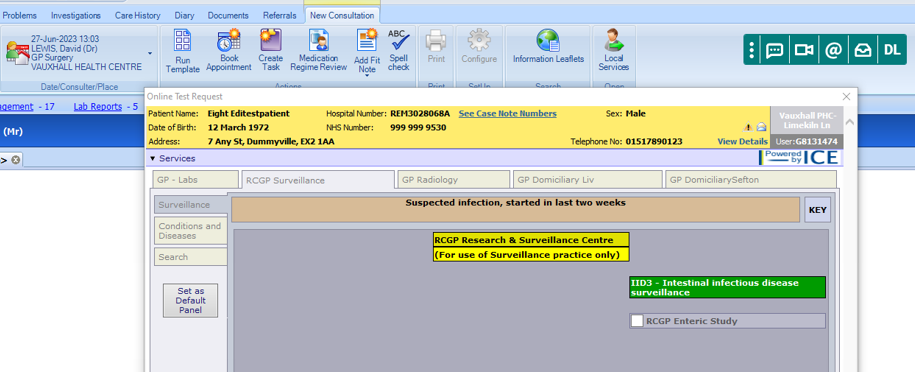


Within that panel the appropriate Study programs will be made available. This will include:

- RCGP Enteric Study
- RCGP Serology Study
- RCGP Virology Study

As part of this configuration, additional information can be made available to offer guidance to the GP. Additional information can be gathered through the use of questions as illustrated below:


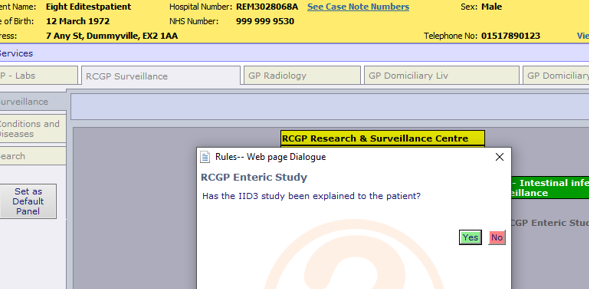
 (In this example, asking if the process has been explained)

The requesting process concludes with the collection of the sample and the ability to add any additional clinical details. A sample label will invariably be printed to allow streamlined reception in the Laboratory


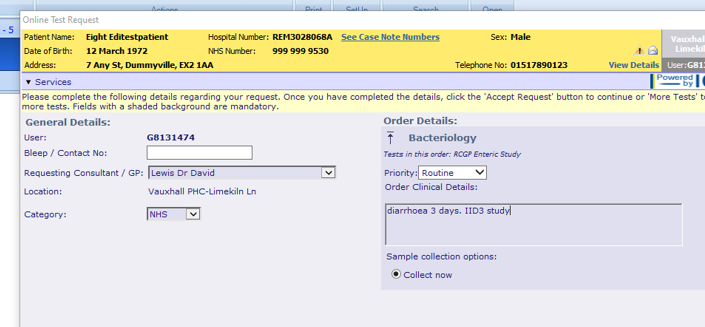
 (Additional Clinical notes have been added)

The sample will then be collected on the routine courier rounds provided by the local Laboratory and the sample transported to specimen reception. The request will have already been received electronically by the local Laboratory. Upon receipt of the sample, the Laboratory will have configured the study as a send-away request. The Laboratory will then book the specimen out for transport to the appropriate performing Laboratory:

- UKHSA – Colindale
- UKHSA – Manchester
- Liverpool Clinical Laboratories

The request will be sent electronically using the X-Lab Labgnostic network and the sample will be transported typically using Royal mail or DX. A part of this process also allows the creation of a shipping manifest allowing visibility of the inbound sample to be available to the performing Laboratory.

The performing Laboratory will receive the request as a message and this will automatically update the Laboratory Information System (LIS) thus negating the requirement for manual request entry. This has the benefit of speeding up the booking in process as well as reducing the occurrence of transcription errors.

Once the required testing has been completed, the result(s) will be created and these are returned to the referring Laboratory via the X-Lab network. This then allows the result to be sent electronically back to the originating GP via the established GPLinks mechanism. This allows the result to be received and included in the patient record.


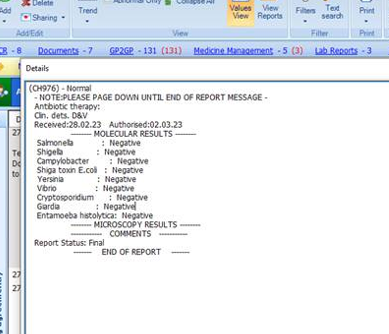
 (Enteric Study results imported into patient record)

An illustration of the full end to end Virology workflow is below:


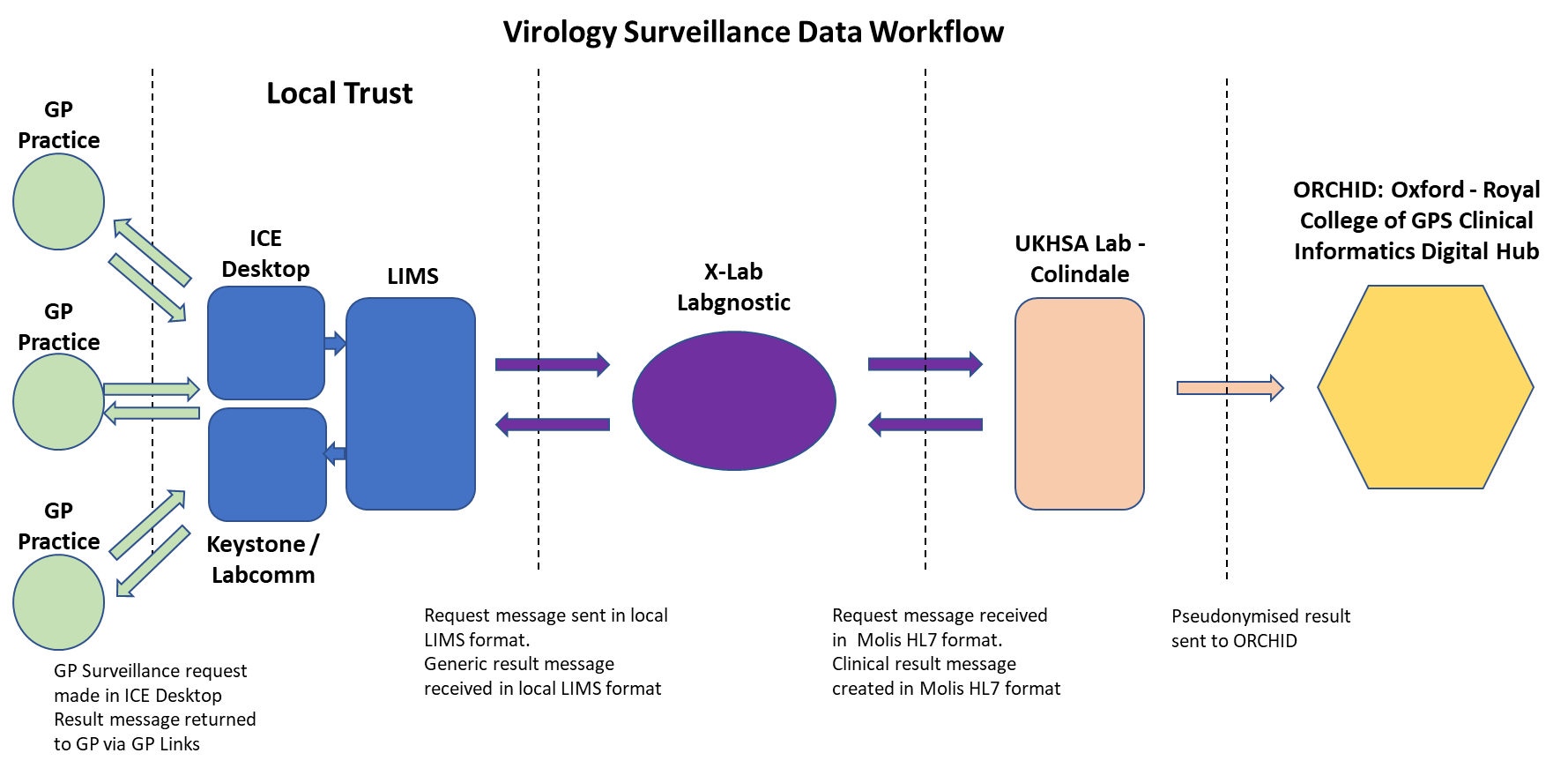

Supplement: Multimedia Appendix 7 [file publichealth_v10i1e52047_app7.docx]
