# Supplementary material for: Postpandemic Sentinel Surveillance of Respiratory Diseases in the Context of the World Health Organization Mosaic Framework: Protocol for a Development and Evaluation Study Involving the English Primary Care Network 2023-2024
Source: JMIR Public Health Surveill. 2024 Apr 3;10:e52047. doi: 10.2196/52047 (PMC11024753; doi:10.2196/52047)
Supplement: Multimedia Appendix 8 [file publichealth_v10i1e52047_app8.docx]

# Multimedia Appendix 8. A protocol for a study using point-of-care testing.

**Enhanced surveillance of children and young adults with acute respiratory infections (ARI) and scarlet fever for Group A Streptococcus, including point of care testing (POCT): English primary care sentinel network pilot (EASTer) study**

Group A Streptococcus (GAS) is associated with respiratory disease, scarlet fever, and occasionally more serious complications. Over the winter period of 2022/23 there was a surge in cases of both scarlet fever and iGAS across England.^[[1]](#footnote-1)^ A proportion of the GAS cases have been invasive (iGAS). Given the high levels of GAS this has led to increased deaths among children.^[[2]](#footnote-2)^ GAS is highly transmissible, and early recognition of scarlet fever and treatment with antibiotics reduces the duration of ill health.^[[3]](#footnote-3),^^[[4]](#footnote-4)^ It also reduced the population prevalence, curbing transmission and preventing serious complications in close contacts.^[[5]](#footnote-5)^ The incidence of disease this winter was a magnitude higher than that seen in 2018, the last recent year with very high levels of infection (Figure 1). In Spring 2023 this has returned to more seasonal numbers, but iGAS remains elevated. This increase in GAS was also reported nationally by UKHSA from clinical disease and laboratory notification data as well as by the RSC in its Weekly Return.^[[6]](#footnote-6)^ The reason for the rise is not known but reduced immunity due to the non-pharmacological interventions across the pandemic may have been important.

We plan to conduct enhanced surveillance of children and young people under the age of 15 years presenting to primary care. We wish to do this to fill important evidence gaps in our understanding of GAS disease and its presentation, meeting evidence gaps identified by UKHSA as critical public health research needs. The UKHSA evidence gaps are labelled iGAS-02 to iGAS-11.

The main evidence gaps we are looking to fill are

- To understand the role of POCT (both molecular and rapid antigen platforms) in rapid diagnosis in the primary care setting (iGAS-04),
- To compare the results of POCT with that from clinical prediction rules (iGAS-02).
- To evaluate what is the utility of clinical prediction scores when combined with POCT in children (iGAS-21)

1. Ledford H. Why is strep A surging - and how worried are scientists? Nature. 2022 Dec;612(7941):603. doi: 10.1038/d41586-022-04403-y. [↑](#footnote-ref-1)
2. Venkatesan P. Rise in group A streptococcal infections in England. Lancet Respir Med. 2022 Dec 19:S2213-2600(22)00507-0. doi: 10.1016/S2213-2600(22)00507-0. Epub ahead of print. [↑](#footnote-ref-2)
3. Cordery R, Purba AK, Begum L, Mills E, Mosavie M, Vieira A, Jauneikaite E, Leung RCY, Siggins MK, Ready D, Hoffman P, Lamagni T, Sriskandan S. Frequency of transmission, asymptomatic shedding, and airborne spread of Streptococcus pyogenes in schoolchildren exposed to scarlet fever: a prospective, longitudinal, multicohort, molecular epidemiological, contact-tracing study in England, UK. Lancet Microbe. 2022 May;3(5):e366-e375. doi: 10.1016/S2666-5247(21)00332-3. Epub 2022 Mar 10. [↑](#footnote-ref-3)
4. Herdman MT, Cordery R, Karo B, Purba AK, Begum L, Lamagni T, Kee C, Balasegaram S, Sriskandan S. Clinical management and impact of scarlet fever in the modern era: findings from a cross-sectional study of cases in London, 2018-2019. BMJ Open. 2021 Dec 24;11(12):e057772. doi: 10.1136/bmjopen-2021-057772. [↑](#footnote-ref-4)
5. Watts V, Balasegaram S, Brown CS, Mathew S, Mearkle R, Ready D, Saliba V, Lamagni T. Increased Risk for Invasive Group A Streptococcus Disease for Household Contacts of Scarlet Fever Cases, England, 2011-2016. Emerg Infect Dis. 2019 Mar;25(3):529-537. doi: 10.3201/eid2503.181518. Epub 2019 Mar 17. [↑](#footnote-ref-5)
6. UK Health Security Agency (UKHSA). Research and analysis: Group A streptococcal infections: report on seasonal activity in England, 2022 to 2023. Updated 30 March 2023. URL: <https://www.gov.uk/government/publications/group-a-streptococcal-infections-activity-during-the-2022-to-2023-season/group-a-streptococcal-infections-report-on-seasonal-activity-in-england-2022-to-2023> [↑](#footnote-ref-6)
